# Radar-Infrared Multi-Scale Bi-Stealth via Optically Transparent Chaotic Coding Metasurface

**DOI:** 10.1007/s40820-026-02294-6

**Published:** 2026-07-20

**Authors:** Yanzhao Wang, Yanzhang Shao, Dan Liu, Zhixuan Hu, Yifei Xu, Huanhuan Gao, Xihong Wang, Xiaogang Su, Fei Ding, He-Xiu Xu

**Affiliations:** 1 Air and Missile Defense College, Air Force Engineering University, Xi’an, 710051 People’s Republic of China; 2 Integrated Circuit Industry College, Wangzheng School of Microelectronics, Changzhou University, Changzhou, 213164 People’s Republic of China; 3 Key Laboratory of Functional Nanocomposites of Shanxi Province, School of Materials Science and Engineering, North University of China, Taiyuan, 030051 People’s Republic of China; 4 School of Electronic Science and Technology, Eastern Institute of Technology, Ningbo, 315200 People’s Republic of China

**Keywords:** Chaotic coding; Radar-infrared bi-stealth, Metasurface, Radar cross section (RCS) reduction, Multi-scale

## Abstract

The rapid development of multispectral detection technology urgently requires the simultaneous suppression of microwave and infrared (IR) signatures. However, conventional strategies suffer from limited functional integration, complex structures, and poor scalability in achieving synergistic control of radar cross section (RCS) and IR radiation characteristics. Herein, we propose a chaotic paradigm combined with a multi-scale strategy to address radar-IR-optical multispectral stealth by using a single-layer coding indium tin oxide (ITO) platform. This architecture covers millimeter-scale representative elements, centimeter-scale phase-coded subarrays, and decimeter-scale meta-arrays, with a direct correlation established between chaotic initial conditions and microwave/IR responses theoretically. Specifically, chaotic coding, a deterministic pseudo-random coding method, is adopted to construct a meta-array inspired by sensitivity of chaotic systems to initial conditions. Tuning chaotic initial parameters enables controllable spatial IR emissivity modulation while preserving broadband intrinsic microwave diffusion due to the multi-wavevector mechanism. For verification, a proof-of-concept metadevice is fabricated, and experimental results manifested a broadband RCS reduction over 10 dB within X/Ku bands (8 ~ 18 GHz) for incident angles up to 45°, with a low IR emissivity below 0.3 and a high optical transmittance of 71.2%. Featuring ultrathin profile (3.35 mm, ~ 0.09 λ_L_), light weight, optical transparency, and facile fabrication, our strategy offers a promising avenue for multi-scale multispectral stealth applications.

**Supplementary Information:**

The online version contains supplementary material available at 10.1007/s40820-026-02294-6.

## Highlights

- A chaotic paradigm combined with a multi-scale strategy is proposed for radar-IR Bi-stealth based on a single-layer indium tin oxide metasurface.
- The metadevice achieves ≥10 dB RCS reduction (8–18 GHz, ≤45°), low IR emissivity (<0.3), and high optical transmittance (71.2%).
- The design features ultrathin profile (3.35 mm), lightweight, optical transparency, and facile fabrication for scalable applications.

**Supplementary Information:**

The online version contains supplementary material available at 10.1007/s40820-026-02294-6.

## Graphical abstract

**Figure:**
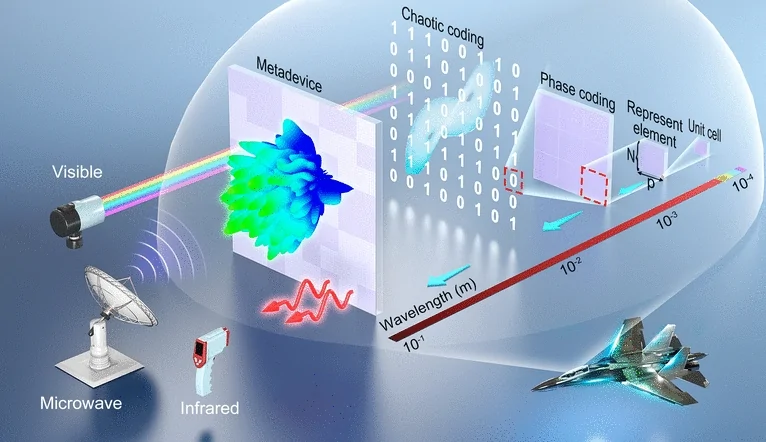

## Introduction

The rapid evolution of multispectral fusion detection systems has rendered conventional single-band stealth technologies obsolete, particularly in surveillance and reconnaissance systems that integrate both radar and infrared (IR) sensing capabilities. Traditional stealth approaches typically suffer from three fundamental limitations. First, the significant wavelength disparity between the microwave (centimeter-scale) and IR (micrometer-scale) regimes necessitates multi-order structural scaling, leading to inherent conflicts in geometric compatibility and design integration [1–3]. Second, the intrinsic contradiction between stealth mechanisms of microwave and IR domains poses a significant challenge in achieving radar-IR bi-stealth. Third, conventional multilayer architectures for multispectral control typically demand sub-micrometer alignment accuracy, posing serious fabrication and scalability barriers for industrial mass production [4]. Currently, radar-IR bi-stealth technologies primarily rely on two methods: functional integration through composite materials [5–9] and multilayer metasurface architecture [10–15]. Although various functional composite materials including magnetic composite aerogels [5, 6], coatings [7], and polymer foams [8] have been widely developed for radar-infrared compatible camouflage, they were restricted to some extent by inherent poor visible-light transmittance in practical applications. Consequently, the development and in-depth research of novel multispectral stealth technologies with transparency are urgently demanded to address the current dilemmas.

Metasurfaces, as planar counterparts of metamaterials, offer significant advantages including low profile, ease of fabrication, and excellent electromagnetic (EM) wave manipulation capability due to their subwavelength feature [16–21]. Leveraging their excellent capability to manipulate amplitude, phase, and polarization of EM waves, metasurfaces have demonstrated revolutionary potential in stealth applications, encompassing microwave radar stealth [22–32], IR camouflage [33–36], and multispectral compatible stealth [37–48]. In general, two physical mechanisms involved amplitude control [22–25] and phase manipulation [26–32] are mainly utilized in radar stealth. In former case, a classic multispectral stealth strategy relies on a multilayer configuration, in which an IR shielding layer is overlaid on top of a microwave absorbing layer. Moreover, realizing broadband microwave stealth often necessitates thick multilayer absorption, which requires additional optimizations [49]. For phase avenue, scattering suppression provides an effective alternative by preventing heat accumulation induced by multilayer absorption, thereby achieving both wideband RCS reduction and low IR emissivity within a single-layer reflective architecture [50, 51]. However, traditional phase-control strategies represented by checkerboard generate regular phase distributions and thus hardly simultaneously meet the requirement for emissivity control in IR band [51]. Most importantly, multi-band compatible stealth applications demand integrated EM response across vastly different wavelength regimes, which remains fundamentally challenging by using low-profile designs due to cross-scale dimensional constraints. Overcoming this bottleneck requires a novel paradigm via innovative multi-scale designs [23], which circumvents inherent drawbacks of multilayer designs, such as interlayer alignment deviation, interfacial EM loss, and complicated manufacturing procedures. Consequently, developing a multi-scale design methodology and establishing a modulation mechanism linking microwave and IR responses have emerged as key challenges for transparent bi-stealth metasurface devices.

**Fig. 1:**
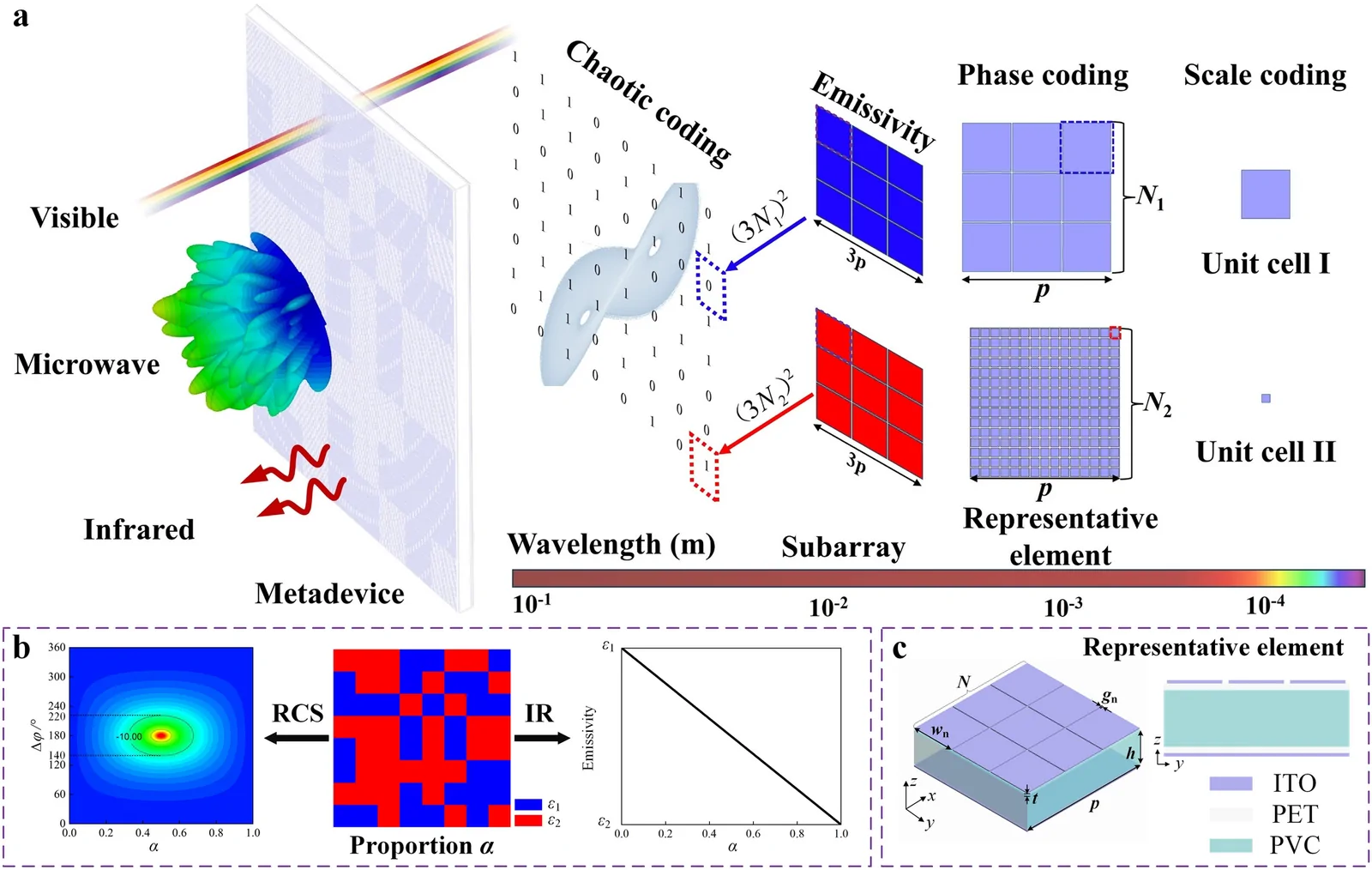
Conceptual illustration of the multi-scale metasurface design for multispectral compatible stealth, integrating microwave scattering suppression and low IR emissivity. **a** Multi-scale design concept integrates a hierarchical architecture featuring millimeter-scale representative elements, centimeter-scale phase-coding subarrays, and decimeter-scale global array configurations. The schematic depicts representative elements of “0” and “1” consisting of different-sized (multi-scale) *N*_1_ × *N*_1_ unit cell I and *N*_2_ × *N*_2_ unit cell II, respectively. **b** Microwave RCS and IR emissivity as a function of the proportion *α*. **c** Schematic view of a representative element

To address above challenges, here we propose a chaotic paradigm and multi-scale strategy to enable microwave-IR bi-stealth by simultaneous modulation of microwave amplitude, phase, and IR emissivity within irregular subarrays determined by chaotic initial parameters. Therefore, the core contribution of this work lies in synergistic innovation at both the system-integration level and design-methodology advance, rather than a new single-band stealth mechanism. Unlike conventional phase-coding metasurfaces composed of identical elements, our multispectral stealth architecture synergistically integrates scale coding (different-sized unit cells) and phase coding (representative elements) to address multi-scale compatibility challenges (Fig. 1). In this multi-scale strategy, the single ITO platform simultaneously synergizes microwave amplitude and phase engineering, while enables IR emissivity tuning via filling-ratio modulation. Building on this concept, we theoretically establish direct correspondence between chaotic initial conditions and resulting microwave and IR responses by introducing a deterministic 1-bit coding paradigm (“0” and “1” representative elements). Therein, a scaling factor *α,* defined as the proportion of “0” elements with emissivity *ε*_1_ to entire metasurface, was utilized to directly link achievable microwave RCS bounds and IR emissivity. As illustrated in Fig. 1b, both microwave RCS and IR emissivity can be expressed explicitly as a function of *α* which enables predictable trade-offs between them without resorting to multi-objective global optimization. A chaotic algorithm was adopted to design *α* and thus finally determined the multi-scale integrated bi-stealth metadevice, facilitating concurrent microwave RCS reduction and low IR emissivity while maintaining optical transparency. Such a chaotic paradigm offers a new avenue for efficient design of multispectral stealth devices.

## Design of Representative Elements

To achieve multispectral compatible stealth, a multi-scale coding metasurface strategy is proposed in Fig. 1a. First, millimeter-scale representative elements with the same period of *p* are divided into *N* × *N* subwavelength identical micrometer-scale unit cells. Next, considering an optimal trade-off among phase-control range and inter-element EM coupling, a 3 × 3 subarray is constructed to realize microwave RCS manipulation at centimeter scale, where the "0" and "1" elements, respectively, encompass (3*N*_1_)^2^ and (3*N*_2_)^2^ different-sized unit cells. Ultimately, at decimeter-scale system level, chaotic algorithm generates coding matrices for pseudo-random metasurface layouts, leveraging the aperiodic characteristics to broaden RCS reduction bandwidth. In addition, proportion *α* is introduced to enable simultaneous control of IR emissivity and microwave RCS.

**Fig. 2:**
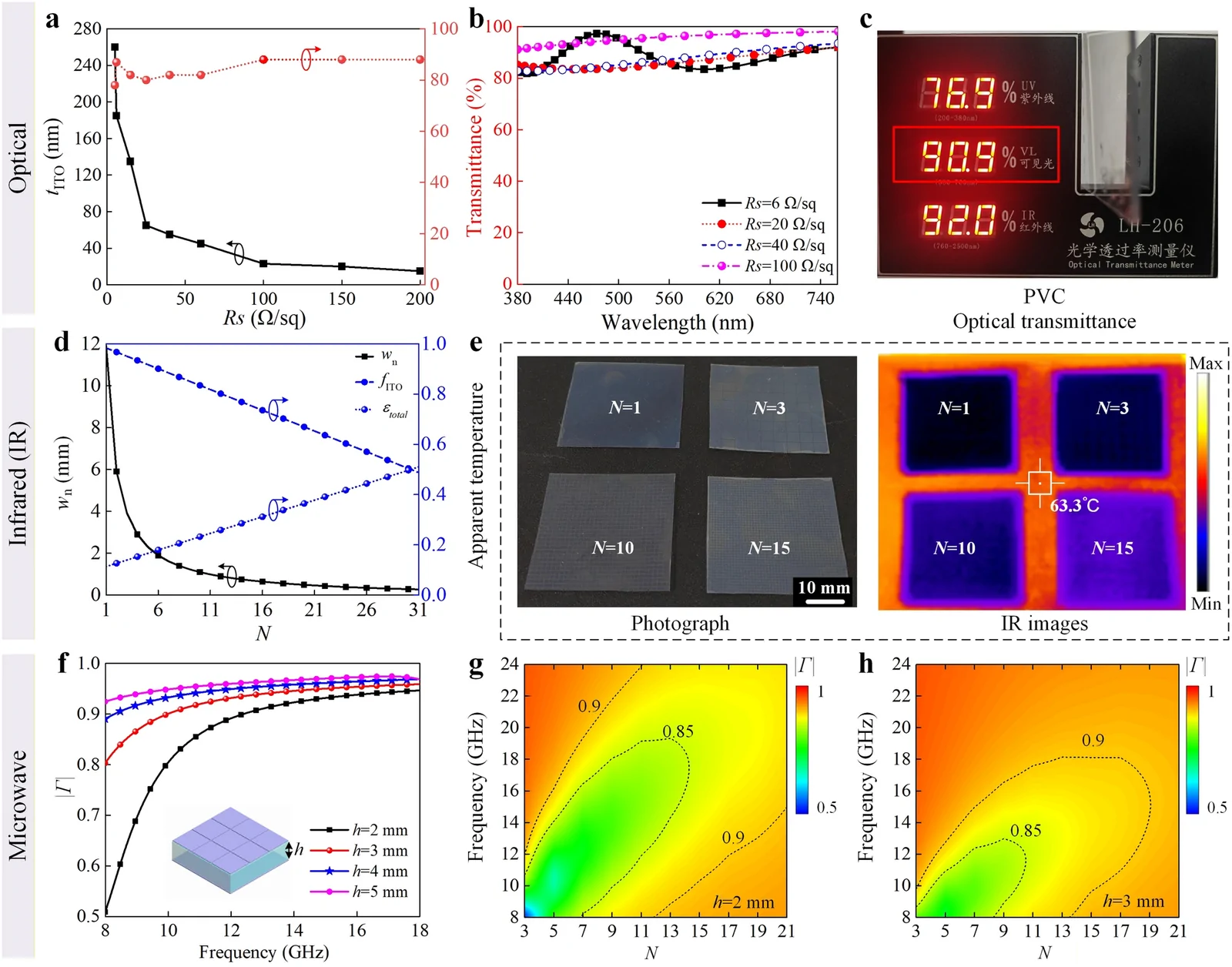
Design and characteristics of the representative element in the optical, IR, and microwave bands. **a** Thickness of the ITO films and the corresponding measured optical transmittance as functions of sheet resistance. **b** Simulated optical transmittance under different sheet resistance. **c** Measured optical transmittance of PVC. **d** Calculated IR emissivity of representative elements with different *N*. **e** Thermal IR images of four representative samples with *N* = 1, 3, 10, and 15. Simulated microwave reflectivity of representative elements with **f** different *h* for *N* = 3 and *N* ranging from 3 to 21 for **g** *h* = 2 mm and **h** 3 mm under *y*-polarization

Different from traditional unit cell-based design, we adopt a multi-scale design paradigm to investigate the characteristics of representative elements. As shown in Fig. 1c, a sandwiched ITO-PVC-ITO element is proposed to achieve cost-effective, lightweight, and optically transparent devices. To characterize the optical performance of ITO films, optical spectra of representative samples with different sheet resistance and film thicknesses are illustrated in Fig. 2a, b, with detailed parameters listed in Table S1. The experimental results indicate that sheet resistance and film thickness are inversely correlated. Conversely, a higher sheet resistance gives rise to degraded IR camouflage performance, as depicted in Figs. S1 and S2. Considering the trade-off among microwave, optical transparency, and IR stealth properties, a 185 nm-thick ITO film with a sheet resistance of *R*_*s*_ = 6 Ω sq^−1^ was ultimately selected as top and bottom layer. For optical transparency, a 3 mm-thick PVC film (*ε*_r_ = 2.45 and tanδ = 0.012) is chosen as middle substrate, which exhibits a measured transmittance of 90.9% (Fig. 2c). Benefiting from the application of ITO thin films and PVC substrates, the metadevice simultaneously achieves optical transparency and mechanical flexibility, demonstrating promising potential for flexible coating applications.

For multi-scale control, the top layer of each representative element is composed of *N* × *N* periodic ITO square patches (Fig. 1). Considering manufacturing accuracy and design convenience, the gap between adjacent patches is fixed at *g*_n_ = 0.1 mm. Consequently, the width *w*_n_ of each unit cell is solely determined by *N* according to (*w*_n_ + *g*_n_) × *N* = *p*. Figure 2d depicts *w*_n_ and the ITO filling-ratio *f*_ITO_ as a function of *N* when *p* = 12 mm, see Table S2 for more details. Although the representative element exhibits a discrete structure macroscopically, ITO behaves as a continuous material with a thickness of 185 nm at the mesoscopic level (Fig. S2). Since ITO patches are patterned on a 175 μm-thick polyethylene terephthalate (PET) substrate, the total IR emissivity can be calculated as $\varepsilon_{total} = \varepsilon_{ITO} f_{ITO} + \varepsilon_{PET} f_{PET}$, where *ε*_ITO_ = 0.1 and *ε*_PET_ = 0.9 denote the IR emissivity of ITO and PET, respectively, and *f*_ITO_ and *f*_PET_ represent filling ratios with *f*_ITO_ + *f*_PET_ = 1. As shown in Fig. 2d, *ε*_total_ increases monotonically with *N* due to the decreased *f*_ITO_ when *g*_n_ is fixed. Therefore, to obtain a lower IR emissivity, it is necessary to enlarge the ITO patch area with a smaller *N*. To further verify IR characteristics of representative elements with different *N*, four samples (*N* = 1, 3, 10, and 15) with the same size of 36 × 36 mm^2^ were fabricated and characterized using an IR spectrometer and a thermal imager (Fig. S1). Correspondingly, the apparent temperature in Fig. 2e also rises with increasing *N*. These results confirm that larger *N* leads to higher IR emissivity, thereby degrading the IR camouflage performance.

For phase-controlled metasurfaces, high reflectivity of the representative element is crucial to microwave performance. Figure 2f illustrates simulated microwave reflectivity as a function of PVC thickness *h* and operating frequency. The calculated thickness-to-bandwidth ratios at various thicknesses with *N* = 3 are 0.43, 0.37, 0.41, and 0.46, respectively, where the bandwidth is defined as the frequency range with a reflection coefficient exceeding 0.9. Based on above analysis, *h* = 3 mm is selected as the optimal thickness with minimum thickness-to-bandwidth ratio in Fig. 2f. Increasing *h* not only enhances reflection amplitude *|Γ|*, but also expands the spectral range where *|Γ|* exceeds 0.9, see Fig. 2g, h. This is attributed to the resonance frequency of representative element shifts to lower frequencies by increasing *h*, which demonstrates an enlarged scattering bandwidth. Furthermore, it exhibits polarization insensitivity due to square-patch’s inherent fourfold rotational symmetry (Fig. S3).

**Fig. 3:**
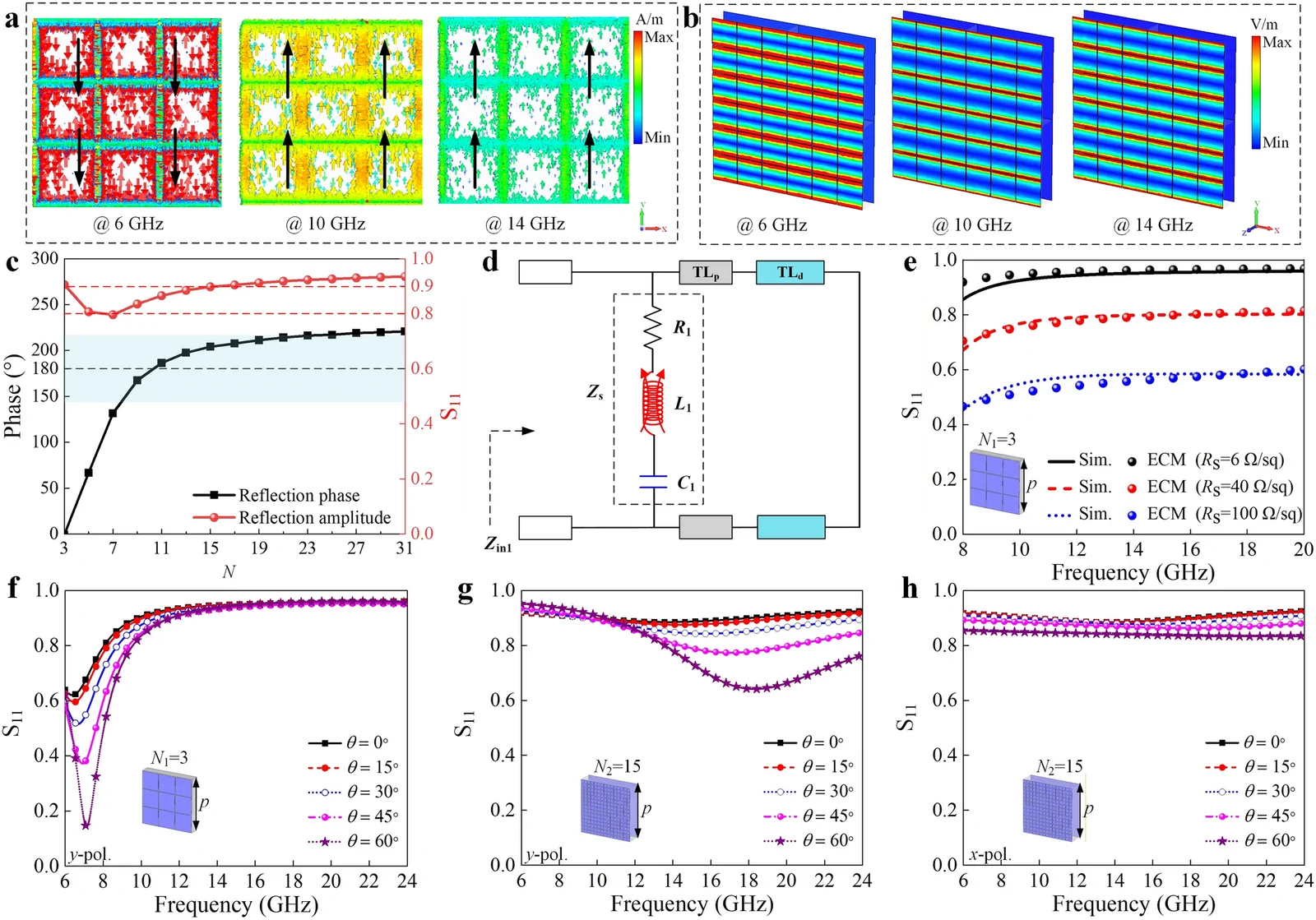
Microwave characterization of the representative element. **a** Surface current and **b** electric field distribution of representative elements at 6, 10, and 14 GHz under *y*-polarization. **c** Reflection coefficient S_11_ and phase with different *N*. **d** Schematic of the ECM. **e** Comparison of S_11_ spectra obtained from the ECM and full-wave simulations for *N*_1_ = 3. **f** Simulated S_11_ for *N*_1_ = 3 with different incident angles. **g**, **h** Simulated reflection amplitude for *N*_2_ = 15 with different incident angles under *y* and *x*- polarizations

Further investigation of reflection amplitude and phase characteristics reveals critical insights into the design mechanism (Fig. 3). As illustrated in Fig. 3a, under *y*-polarized illumination, oscillating surface currents are induced along electric field direction on the square ITO patch, with the current density primarily concentrated in central region. The time-varying currents generate a circulating magnetic field around the patch, establishing an equivalent inductance (Fig. S4). Simultaneously, strong electric fields are concentrated at two ends of the square patch, constituting an equivalent capacitance (Fig. 3b). Figure 3c illustrates *|Γ|* and phase *φ* variations at 10 GHz as *N* increases from 3 to 31. *φ* increases significantly with *N* at the beginning, while both the phase and amplitude gradually stabilize at higher *N* due to reduced dimensional sensitivity. For coding metasurface, the representative "0" and "1" elements are required to exhibit amplitudes above 0.9 and a relative phase difference of 180°. Two representative cases (*N*₁ = 3 and *N*₂ = 15) are therefore selected for detailed analysis. To further study the influence of *N* on reflection amplitude, a theoretical analysis based on transmission-line theory is performed by establishing a simplified equivalent circuit model (ECM), see Fig. 3d. Here, the ITO patch film is modeled by a series *RLC* circuit, where the equivalent resistance is calculated as *R*_1_ = *R*_*s*_*·S*_*unit*_ / *S*_*eff*_, with *S*_*unit*_ and *S*_*eff*_ representing the total area of representative element and the effective area of ITO square patches, respectively. Then, the reflection coefficient *Γ* can be derived from *Γ* = (*Z*_in_ − Z₀) / (*Z*_in_ + Z₀) with its phase *φ* = arg(*Γ*). Here, *Z*_in_ = *R*₁ + j(*ωL*₁) − j(*ωC*₁)^−1^ indicates equivalent input impedance of representative element, and Z₀ = 377 Ω is free-space impedance. Furthermore, the relationship between stealth performance and surface impedance is governed by Z_in_ via equivalent circuit model. Therefore, tailoring the geometric parameter *N* of representative elements allows for direct modification of its circuit parameters. This variation, in turn, alters its input impedance *Z*_in_, which ultimately determines the reflection magnitude |*Γ*| and phase *φ*. The equivalent circuit parameters were retrieved for different *N* according to the empirical formula [52] (see Derivation of the ECM in Supporting Information). For verification, Fig. 3e compares theoretical results based on idealized ECM with full-wave simulations for *N*₁ = 3 (*C*₁ = 0.216 pF, *L*₁ = 1.806 pH, and *R*₁ = 6 Ω), where good agreement is observed with minor deviations at lower frequencies primarily due to neglected mutual coupling effects in ECM.

Figure 3f further illustrates S_11_ as a function of frequency at various incident angles under *y*-polarization. It is evident that S_11_ remains nearly constant above 10 GHz and consistently stays above 0.9 when *N*_1_ = 3. Noted that resonance effects cause a decrease in reflection amplitude with increased incident angles near 8 GHz. Therefore, to minimize the impact, it is necessary to ensure the resonant frequency is far away from the operating band. As illustrated in Fig. 3g, h, the representative element with *N*_2_ = 15 maintains high reflectivity across incident angles up to 45°, demonstrating its angular stability.

## Results and Discussion

### Design of Chaotic Coding Metasurface

The far-field scattering pattern of a metasurface is governed by the Fourier transform of its reflection-coefficient distribution $r(x,y) = e^{i\varphi (x,y)}$ [31]. Therefore, we need to select such a *φ*(x, y) distribution that can make scattering pattern as uniform as possible to achieve the best wave-diffusion performance. Here, the reflection-coefficient distribution r(x,y) is modeled as a two-dimensional wide-sense stationary stochastic process. Based on Wiener–Khinchin theorem, the Fourier transform of its spatial autocorrelation function R(x,y) is proportional to far-field scattering pattern. Uniform far-field scattering requires a spatially isotropic and sharply peaked R(x,y) (ideally a delta function), implying that the reflection coefficient is spatially uncorrelated. The distribution of scattered wavevectors is governed by the spatial architecture of coding metasurfaces. By their intrinsic nature, chaotic coding sequences possess such delta-like autocorrelation and spatially uncorrelated characteristics, thus enabling the distribution of scattered wavevectors to be highly uniform and stochastic.

**Fig. 4:**
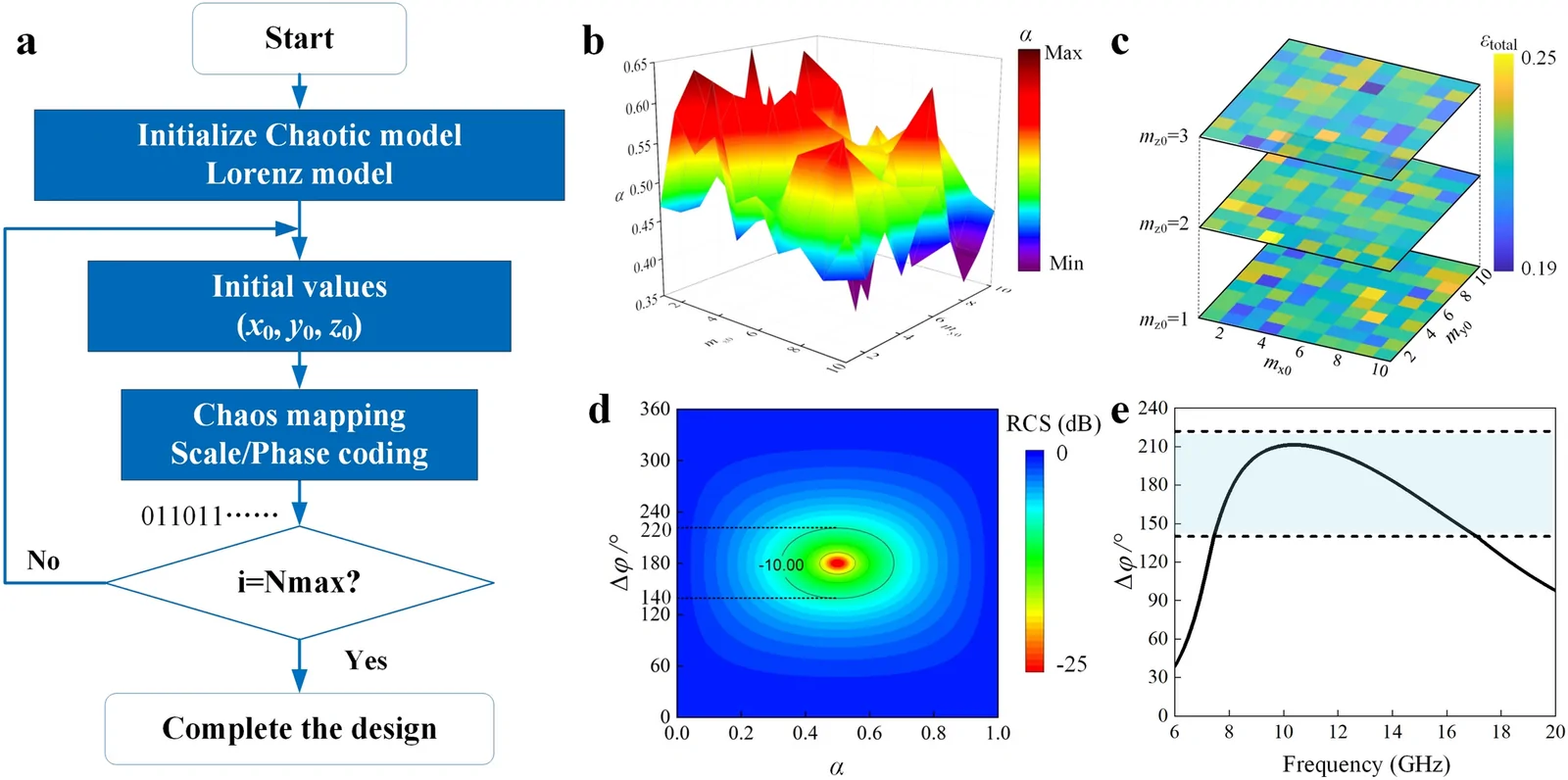
Chaotic coding algorithm for the radar-IR bi-stealth metasurface design. **a** Design flowchart of the chaotic coding algorithm based on the Lorenz model, where binary coding is utilized to arrange reflection phases correlated with different spatial scales. Theoretical IR characteristics of the chaotic coding metasurface with different initial values: **b** scaling factor *α* for *m*_z0_ = 1 and **c** *ε*_total_ under varying initial values. Theoretical microwave characteristics of chaotic coding metasurface: **d** relationship among RCS, *α*, and phase difference Δ*φ*; **e** phase difference spectrum Δ*φ* of two representative elements

Conventional coding metasurfaces with checkerboard configurations inherently suffer from a fixed filling ratio of 50% in standard binary case, which severely constrains their adaptability for IR stealth applications. To overcome this limitation, a deterministic chaotic algorithm is proposed to generate coding patterns. Leveraging the initial-condition sensitivity of nonlinear chaotic systems, it effectively generates deterministic pseudo-random sequences. By varying the initial parameters, the algorithm produces diverse pseudo-random distributions that enable deterministic control over both spatial and spectral characteristics. As depicted in Fig. 4a, the design procedure mainly consists of four main steps. Firstly, an appropriate chaotic model is selected as the core dynamic system. Here, the Lorentz model, one of the most representative chaotic systems, is employed for chaotic coding. The specific equations are expressed as

1 $$ \left\{ {\begin{array}{*{20}l} {\frac{{dm_{x} }}{dt}} \hfill & { = \sigma (m_{y} - m_{x} )} \hfill \\ {\frac{{dm_{y} }}{dt}} \hfill & { = \rho m_{x} - m_{y} - m_{x} m_{z} } \hfill \\ {\frac{{dm_{z} }}{dt}} \hfill & { = m_{x} m_{y} - \beta m_{z} } \hfill \\ \end{array} } \right. $$

where *σ* is the Prandtl’s constant, *ρ* is the Rayleigh’s constant, and *β* is the directional ratio. The typical parameter set (*σ* = 10, *β* = 8/3, and *ρ* = 28) leads to a well-known chaotic regime in the Lorentz system. Secondly, the initial conditions (*m*_x0_, *m*_y0_, *m*_z0_) need to be determined. Thirdly, by numerically integrating Eq. (1) over Nmax iterations with a time step of Δ*t* = 0.01 s, a series of chaotic states is generated. The mean values of these states, defined as *φ*_m_ = (*m*_x_ + *m*_y_ + *m*_z_) / 3, are then mapped to binary codes “0” and “1,” corresponding to reflection phases of 0 and π, respectively. Finally, these binary phase codes are spatially arranged to form meta-array, completing chaotic coding metasurface. Moreover, the metasurface configuration is uniquely determined once initial values are established, thereby significantly reducing design complexity and computation time.

The proposed chaotic coding metasurface overcomes the inherent limitations of conventional checkerboard-patterned designs by introducing tunable IR emissivity through initial-condition sensitivity. According to the numerical homogenization method, the overall IR emissivity is jointly determined by the macroscopic arrangement and microstructural features. Therefore, the equivalent emissivity can be expressed as the average emissivity of all representative elements. For a binary coding metasurface, the scaling factor *α* denotes the proportion of "0" element, and the total emissivity of the metasurface *ε*_total_ is calculated as$\varepsilon_{total} = \varepsilon_{1} \alpha + \varepsilon_{2} (1 - \alpha )$, where *ε*_1_ and *ε*_2_ represent the emissivity of "0" (*N*_1_ = 3) and "1" (*N*_2_ = 15) elements, respectively, whose theoretical values are *ε*_1_ = 0.14 and *ε*_2_ = 0.30. Noted that above equivalent IR emissivity obtained by linear weighting of isolated samples affords a simplified engineering approximation due to thermal diffusion and near-field radiative heat transfer. Nevertheless, our approximation is reasonable for design since randomly distributed chaotic coding pattern further suppresses localized temperature gradients. Following the chaotic coding design, the IR emissivity and the scaling factor *α* with varying initial conditions are investigated in Fig. 4b. Specifically, theoretical analysis with a fixed *m*_z0_ = 1 reveals significant variations in *α* (0.34 ~ 0.65) under different (*m*_x0_, *m*_y0_) values. To further examine IR emissivity range of this method, Fig. 4c presents calculated *ε*_total_ for 300 distinct initial conditions with *m*_z0_ = 1, 2, and 3. As expected, *ε*_total_ varies significantly across different initial values, confirming the strong sensitivity of the chaotic coding metasurface to its initial state. As illustrated in Fig. 4c, the maximum emissivity reaches 0.25 (*α* = 0.296), while the minimum emissivity is 0.19 (*α* = 0.703), demonstrating the tunable IR performance of the design.

For microwave performance, the backward RCS reduction *σ*_r_ of a binary coding metasurface can be calculated as:

2 $$ \sigma_{r} = 10\log \left[ {\alpha A_{1} e^{{j\varphi_{1} }} + (1 - \alpha )A_{2} e^{{j\varphi_{2} }} } \right]^{2} $$

where* A*_1_ and *A*_2_ represent reflection amplitudes of representative elements "0" and "1," respectively, and *φ*_1_ and *φ*_2_ are corresponding reflection phases. When *A*_1_ = *A*_2_ = 0.9, *N*_1_ = 3, and *N*_2_ = 15, RCS is mainly determined by *α* and the phase difference (Δ*φ* = *φ*_2_—*φ*_1_), see Fig. 4d. To obtain an RCS below − 10 dB, the widest phase difference range occurs at *α* = 0.5, satisfying 140° < Δ*φ* < 222°, which aligns with the phase cancelation condition in checkerboard metasurfaces. Furthermore, the allowable phase difference becomes more stringent as the ratio disparity increases; for instance, phase difference must satisfy 158° < Δ*φ* < 204° to achieve an RCS reduction of 8 dB at *α* = 0.3 or 0.7. Similarly, when Δ*φ* = 180°, the maximum allowable ratio difference corresponds to a range of 0.33 < *α* < 0.68 for achieving a 10 dB RCS reduction. Figure 4e further illustrates the frequency-dependent variation of phase difference for representative elements "0" and "1." The theoretical requirements are well satisfied within 7.4 ~ 17.1 GHz, confirming broadband RCS reduction capability across a wide frequency range. To illustrate the intrinsically scattering characteristics of this method, we compare the spatial autocorrelation properties of chaotic coding with that of conventional checkerboard coding method. As shown in Fig. S5, the chaotic coding metasurface achieves a completely random and uncorrelated phase distribution, thereby generating a highly uniform far-field scattering pattern. Fig. S6 further illustrates the phase distributions and corresponding theoretical scattering patterns of 2-bit and 3-bit coding metasurfaces, confirming that the approach is readily scalable beyond the binary coding scheme (Supplementary Text S1).

**Fig. 5:**
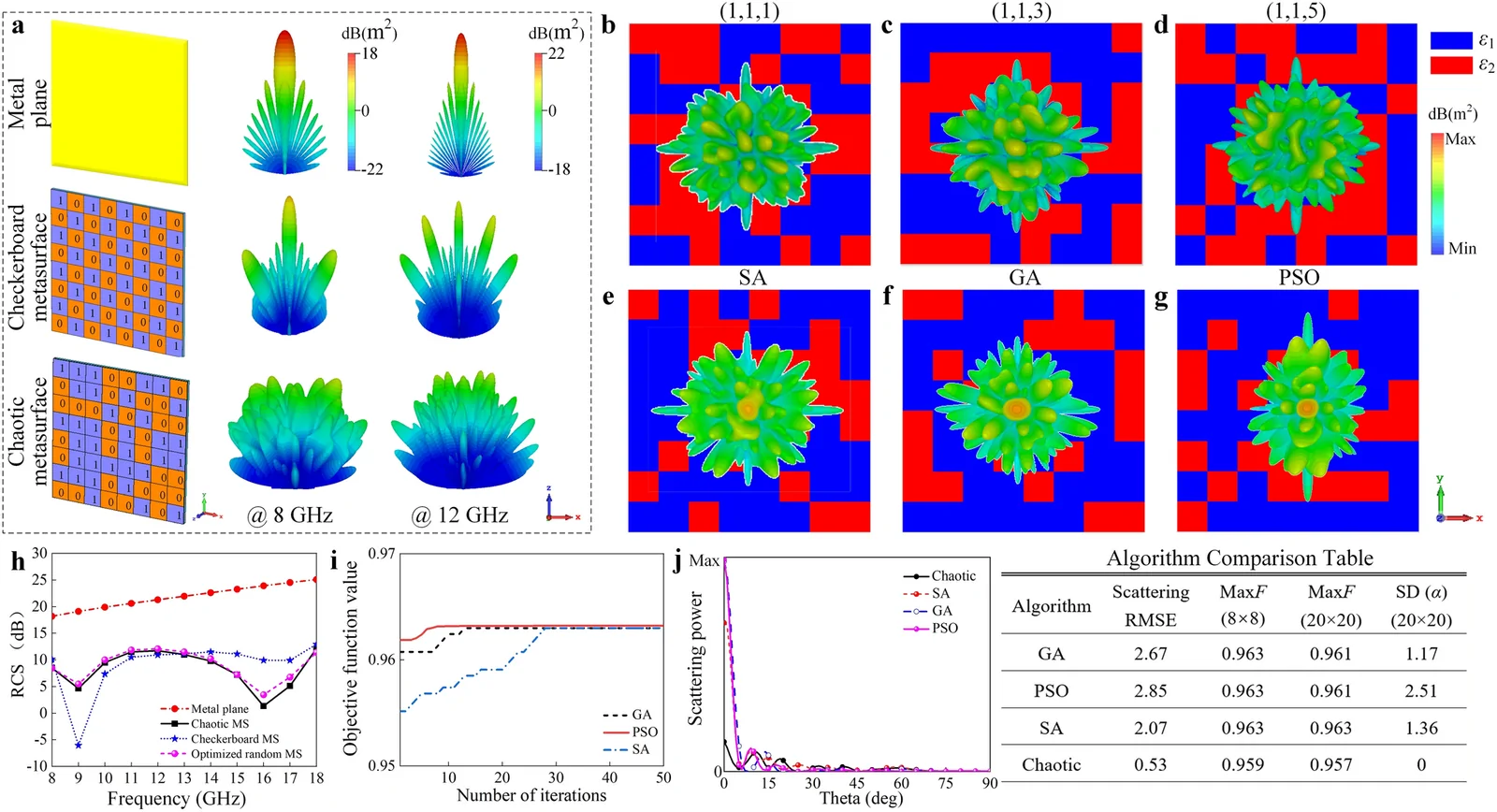
Advantages of the proposed chaotic coding metasurface compared to conventional approaches based on different algorithms. **a** Comparison of simulated microwave far-field patterns among the proposed chaotic coding metasurface, a conventional checkerboard metasurface, and an equal-sized metal plane at representative frequencies of 8 and 12 GHz. Comparison of microwave and IR characteristics at 10 GHz for chaotic coding metasurfaces initialized with **b*** m*_x0_ = 1, **c** *m*_x0_ = 3, and **d*** m*_x0_ = 5 while keeping *m*_y0_ = *m*_z0_ = 1. Metasurfaces optimized using **e** simulated annealing (SA), **f** genetic algorithm (GA), and **g** particle swarm optimization (PSO) algorithms, respectively. **h** Comparison of the RCS for a metal plane, the proposed chaotic coding metasurface, a conventional checkerboard metasurface, and an optimized random metasurface under normal incidence. **i** Comparison of fitness evolution curves and the corresponding optimized results obtained using different algorithms. **j** Comparison of scattering powers obtained for different metasurfaces of (**b**, **e**–**g**) at 10 GHz. Comparison table of scattering RMSE, MaxF (8 × 8 and 20 × 20 subarrays), and SD(*α*) from 50 independent theoretical runs. Here, SD is calculated as $SD(\alpha ) = \sqrt {\frac{1}{n}\sum\limits_{i = 1}^{n} {(\alpha_{i} - \overline{\alpha })^{2} } }$, which illustrates the algorithm’s stability across multiple trials. A lower SD indicates a greater result consistency

Based on aforementioned design principles, we designed a chaotic coding metasurface with an initial condition of (1, 1, 1) and 8 × 8 subarrays, as depicted in Fig. 5a. Different from traditional random encoding, the Lorenz chaotic system can generate ordered and controllable chaotic sequences to achieve uniform diffusive scattering. To verify the superiority of our strategy, comparative simulations were conducted against an equal-sized metal plane and a conventional checkerboard metasurface. Their corresponding far-field scattering patterns at 8 and 12 GHz are presented in Fig. 5a. Remarkably, the proposed chaotic coding metasurface achieves omnidirectional diffusive scattering, which effectively suppresses RCS to 4.93 dB via non-periodic random phase distribution introduced by chaotic paradigm. Notably, the far-field scattering patterns are polarization-independent at normal incidence.

To verify the generality and robustness of our strategy, several metasurfaces with distinct initial values including (1, 1, 1), (3, 1, 1), and (5, 1, 1) were designed and numerically compared with metasurfaces optimized using SA, GA, and PSO algorithms according to the following targeted function (Fig. 5b–g).

3 $$ \max F(X) = w_{1} \cdot \frac{{\sum\nolimits_{i = 1}^{n} {RCS} (f_{i} ,X)}}{n} + w_{2} \cdot \frac{{S_{ITO} (X)}}{S} $$

Here, $X = \{ x_{1} ,x_{2} , \ldots ,x_{64} \} (x_{j} \in \{ 0,1\} ,j = 1,2, \ldots ,64)$ represents the binary coding matrix to be optimized. The term $\sum\nolimits_{i = 1}^{n} {RCS} (f_{i} ,X)$ quantifies the microwave stealth performance by calculating the sum number of frequency points within 8–18 GHz for which the RCS satisfies RCS <  − 10 dB, corresponding to the coding matrix X. Meanwhile, the ratio S_ITO_(*X*)/S denotes the duty cycle of ITO determined by matrix X, which characterizes the corresponding IR stealth performance. In the optimization, the weighting coefficients were set as *w*_1_ = *w*_2_ = 0.5, reflecting the equal importance of microwave and IR stealth performance. In IR emissivity maps, blue and red regions correspond to “0” and “1” elements with *ε*_1_ = 0.14 and *ε*_2_ = 0.30, respectively. The corresponding α/*ε*_total_ values for the six metasurfaces were 0.47/0.22, 0.54/0.21, 0.50/0.22, 0.63/0.20, 0.66/0.19, and 0.66/0.19, respectively. Notably, compared to metasurfaces optimized by conventional algorithms in Fig. 5e–g, our chaotic coding metasurfaces exhibit diffuse and uniformly distributed lobes in the microwave far-field patterns, mainly attributed to the spatially incoherent phase distribution. To illustrate advantages of our strategy, we quantitatively compare the scattering performance of metasurfaces (Fig. 5h-j). Figure 5j depicts how the integrated scattering powers vary against the angle of theta. As is shown, the energy scattered by our chaotic metasurface is greatly homogenized in the *θ*-plane, whereas sharp beams are clearly observed for other algorithms. The corresponding root mean square error (RMSE) is calculated as $RMSE = \sqrt {\frac{1}{n}\sum\nolimits_{i = 1}^{n} {(y_{i} - y_{\min } )^{2} } }$ (Fig. 5j). The lowest RMSE confirms that our metasurface supports much better isotropic scattering performance than other designs. The table also lists the fitness values obtained from 8 × 8 and 20 × 20 array configurations, along with the standard deviation (SD) of *α* computed over 50 independent theoretical runs. Notably, our proposed metasurface exhibits the lowest RMSE of 0.53 and exceptional stability with SD(*α*) = 0, while maintains near-optimal fitness values (maxF≈0.96) comparable to those of optimized designs. Importantly, such performance is achieved without optimization, significantly reducing computational overhead, which is attributed to a deterministic pseudo-random method driven solely by the predefined initial conditions. In contrast, optimization algorithms typically produce varied *α* across repeated executions due to inherent randomness in their search mechanisms. This deterministic stability makes the method particularly suitable for applications requiring high phase consistency such as reconfigurable intelligent surfaces. Furthermore, the characteristics of the extended arrays including 20 × 20 and 30 × 30 subarrays presented in Fig. S7 further validate the effectiveness of our proposed method, and the performance advantages become increasingly pronounced as the array scale expands.

### Experimental Verification

**Fig. 6:**
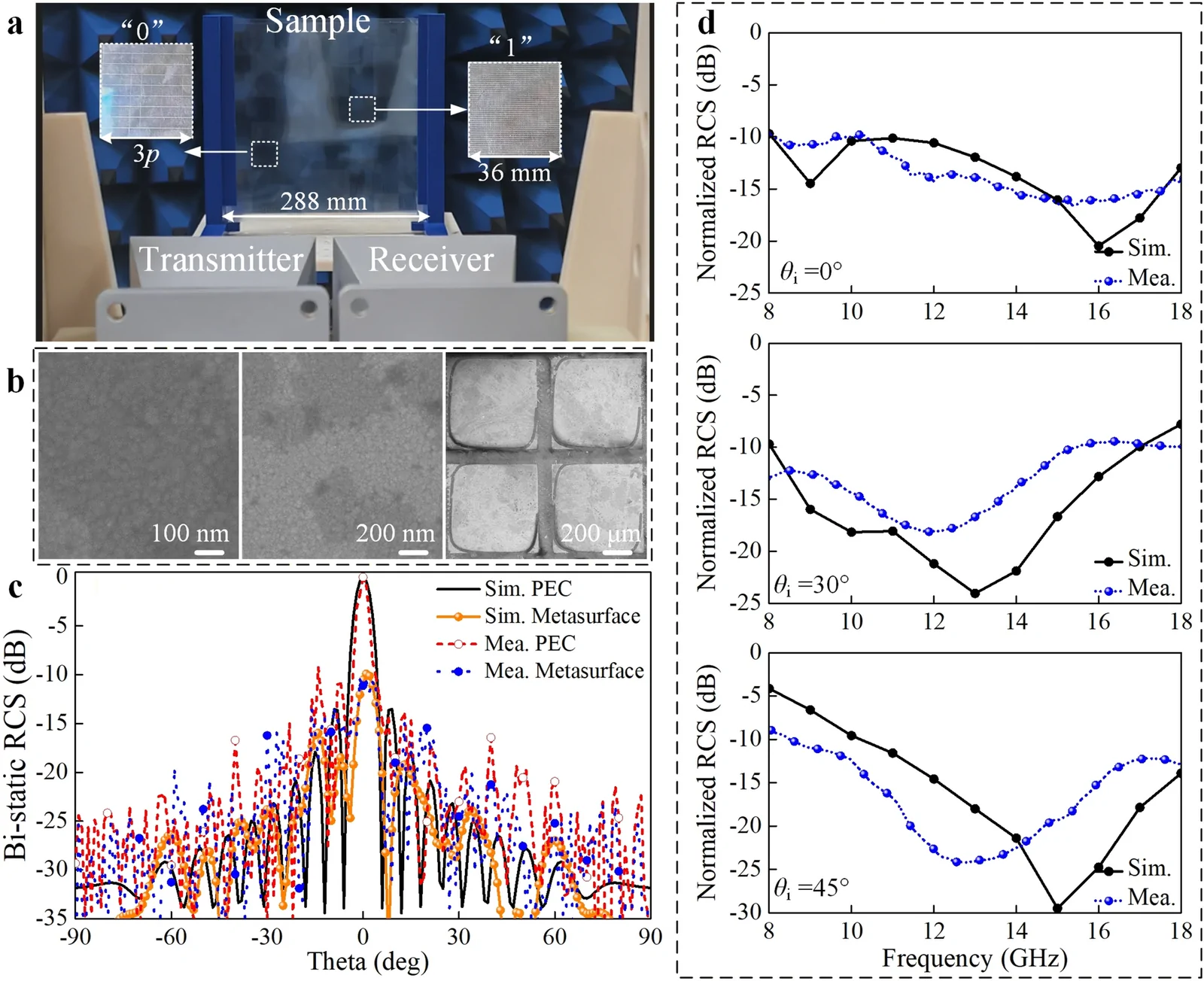
Experimental microwave characterization of the fabricated bi-stealth metadevice. **a** Fabricated metadevice sample and enlarged views of “0” and “1” elements. **b** SEM image of ITO (100 nm and 200 nm) and representative element (200 μm). **c** Comparisons of the simulated and measured far-field bistatic RCS results of the proposed metasurface and a metal plate reference at 10 GHz under *y*-polarized polarization. **d** Simulated and measured normalized RCS of the proposed metasurface with incident angles of 0°, 30°, and 45°, where all RCS values are normalized against the reference metallic plate of the same size

For experimental verification, a sample was fabricated with initial values of (1, 1, 1). An ITO film with a total size of 288 × 288 mm^2^ was deposited on a PET film through magnetron sputtering. The fabricated sample and experimental setups for bistatic radar RCS measurements are shown in Figs. 6a and S9, respectively. Moreover, the inset also depicts enlarged views of the “0” and “1” representative elements, each composed of 3 × 3 elements arranged periodically, resulting in an overall size of 36 mm × 36 mm. Figure 6b presents ITO surface morphologies obtained via scanning electron microscopy (SEM). It is observed that ITO possesses obvious fine particles with uniform size, and the representative elements are periodically arranged with a spacing of 100 μm. Here, ITO and PVC materials are selected for their excellent comprehensive properties. More details are presented in Supplemental Document. Figure 6c compares the measured bistatic RCS at 10 GHz between the fabricated sample and an equal-sized metal plate under *y*-polarized incidence. The experimental results are in good agreement with simulations, demonstrating an RCS reduction of more than 10 dB in the normal-incidence direction. Moreover, the measured results at 8 ~ 18 GHz under x-polarization are presented in Fig. S10. By redistributing the scattered energy into multiple sidelobes, the chaotic coding metasurface embodies the intrinsic operating principle of scattering-based stealth, distinct from absorption-based approaches. As a result, the peak bistatic RCS is substantially reduced compared with that of a metallic reference and a conventional checkerboard metasurface. Furthermore, the resulting sidelobes are diffuse and spatially incoherent, which is advantageous in practical radar detection scenarios relying on strong specular returns. Comparison between measured and simulated RCS is further illustrated in Fig. 6d under different incident angles. Here, all RCS values are normalized to the bare metallic plate of identical size. When the EM wave is incident at angles up to 45°, the RCS reduction of more than 10 dB is maintained within 8 ~ 18 GHz, corresponding to a fractional bandwidth of 76.9%. Minor deviations between measurements and simulations are attributed to environmental influence and system imperfections during measurement. Nevertheless, these effects are minimal, confirming that the proposed chaotic coding metasurface exhibits broadband RCS reduction and wide-angle operational stability. To investigate mechanical stability, the simulation and measurement are conducted in Figs. S11-S13 regarding bending and friction. As is shown, the device still maintains acceptable stealth performance under conformal and partially damaged conditions.

**Fig. 7:**
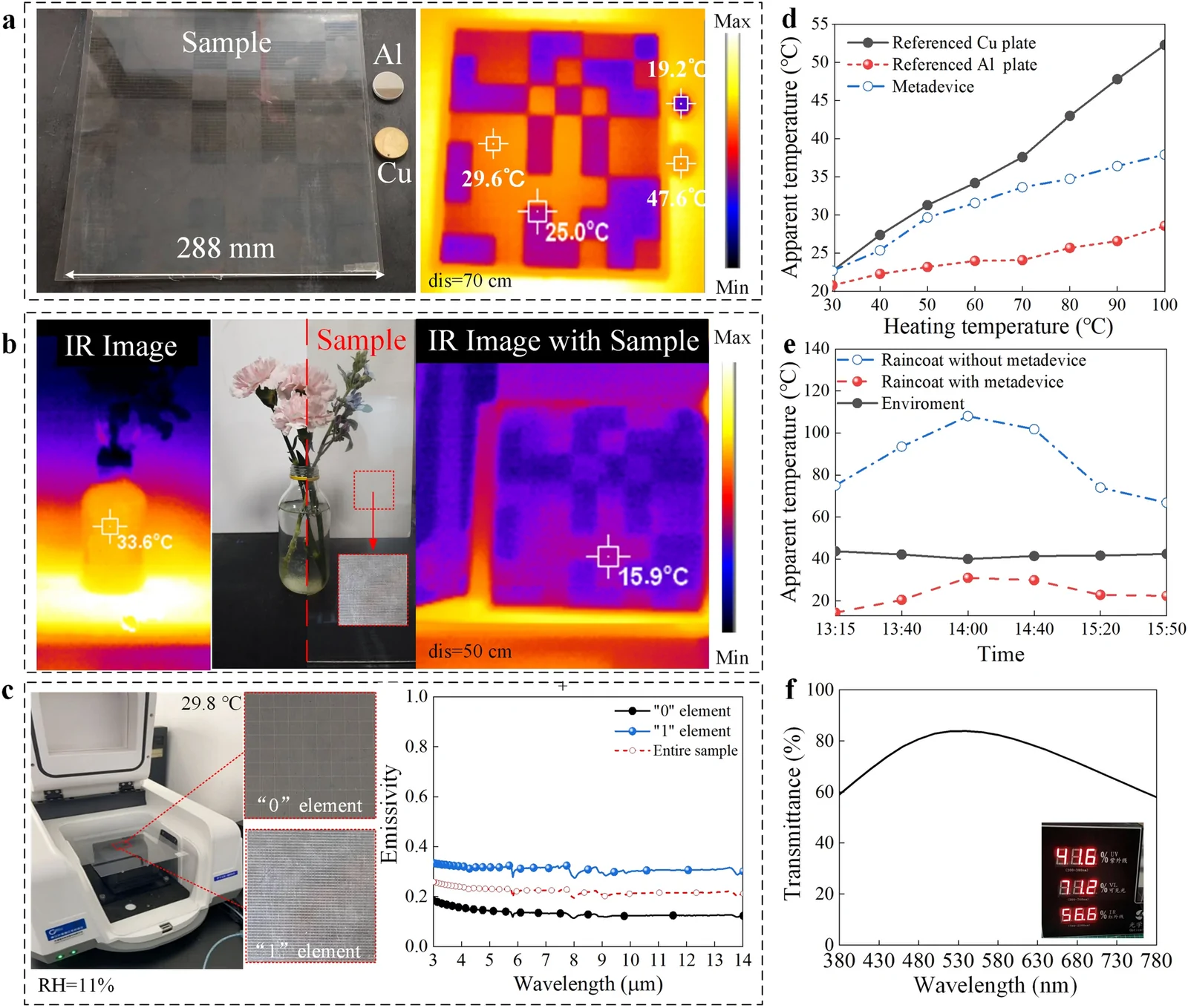
Experimental IR characterization of the proposed bi-stealth metadevice. **a** Photograph and IR image of the fabricated metadevice sample and referenced Cu and Al circular plates with known emissivity. Here, the distance between the sample and thermal imager is 70 cm. **b** Comparison of IR images without (left) and with (right) the sample. Here, the distance between the sample and thermal imager is chosen as 50 cm. **c** Measured IR emissivity of elements “0” and “1” samples with *N* = 3 and 15. **d** Variations of apparent temperature under different heating temperatures. **e** Comparison of apparent temperature of a raincoat with and without the metadevice sample attached. **f** Measured transmittance of the proposed metadevice

Finally, the IR stealth characteristics of the fabricated metadevice were systematically evaluated. As shown in Fig. 7a, the experiment was performed using a heating plate maintained at a constant temperature of 65 °C to assess the thermal radiation performance. The measurement was carried out under environmental temperature of 29.8 °C and relative humidity of 11%. The chaotic metadevice sample was compared with standard metallic references, namely aluminum (Al: *ε*_AL_ = 0.05) and copper (Cu: *ε*_Cu_ = 0.45). Thermal images captured using an IR thermal imager (Smart Sensor ST9450) revealed apparent temperatures of 19.2 °C and 47.6 °C for the Al and Cu plates, respectively. In contrast, the apparent temperatures of “0” and “1” elements were measured to be 25.0 and 29.6 °C, respectively, confirming their effective IR suppression capability. According to the Stefan–Boltzmann law, when the apparent temperature is identical, the emissivity of the sample with a lower radiation temperature is reduced. Consequently, the equivalent emissivity of the metadevice was calculated to be approximately 0.23, which is very close to the theoretical result, thereby satisfying IR stealth requirements.

To further comprehensively assess its IR stealth performance alongside optical transparency, the sample was positioned in front of a transparent vase (middle panel of Fig. 7b). The outline of the flowers behind the sample remained clearly visible. To simulate a real-world scenario, the water in the vase was heated to 33 °C (approximately simulating human thermal radiation) using a bottom heating plate. Despite occlusion by the sample, a clear outline of the flower remained visible in the left panel of Fig. 7b. To quantitatively characterize IR spectra of the metadevice, Fourier transform infrared spectroscopy (FTIR) was performed on the representative “0” and “1” elements. Both elements maintain low infrared emissivity across 3–14 μm (Fig. 7c). Additionally, the measured apparent temperature decreased to 15.9 °C after sample occlusion, closely matching the background temperature of 16.2 °C. These results demonstrate that the designed metasurface exhibits superior IR stealth capability while maintaining excellent optical transparency. The thermal stability of the sample was further investigated under different heating conditions. Owing to the inherent thermal limitation of the PET substrate, the maximum allowable temperature was 120 °C. Therefore, apparent temperatures of Cu, Al, and metadevice sample were measured when the heating temperature varied from 30 to 100 °C (Fig. 7d). Furthermore, outdoor experiments were conducted to evaluate temperature variations (Figs. 7e and S14). In this test, a camouflage raincoat served as the background layer, and the sample was positioned on top. The apparent temperature distributions were compared with and without the sample under peak sunlight at noon, when the ambient radiation temperature remains stable at approximately 42 °C. To further investigate the optical transmittance of the proposed metadevice, we measured its optical performance using an optical transmittance meter. As shown in Fig. 7f, the transmittance was measured in 380–780 nm wavelength range, the transmittance of entire metadevice based on chaotic-coded metasurface yielded a value of 71.2%.

Table 1 provides a detailed and comprehensive comparison with several previously reported microwave-IR bi-stealth metasurfaces to illustrate clear advantages of proposed metadevice. As summarized in Table 1, the proposed metadevice exhibits low IR emissivity, high transparency, and achieves broadband RCS reduction without complex optimization algorithm. Moreover, in contrast to absorption-based methods, scattering-driven strategy minimizes conductive ITO layers. Compared to checkerboard case, our metadevice exhibits superior stealth performance even at 45° oblique incidence.

**Table 1:** Comparison of radar-IR compatible stealth performance among different metasurfaces

Reference	[44]	[45]	[14]	[29]	[50]	[51]	This work
Optical transmissivity	33%	30%	51.7%	60%	√	56%	71.2%
Thickness	0.165 λ_L_	0.10 λ_L_	0.08 λ_L_	0.10 λ_L_	0.06 λ_L_	0.08 λ_L_	0.09 λ_L_
Bandwidth (GHz)	1.5–9 (142.9%)	7.7–18 (80.1%)	7.6–16.4 (73.3%)	8.7–32 (114.5%)	7.5–17 (77.6%)	7.4–13.4 (57.6%)	8–18 (76.9%)
Thickness-to-bandwidth ratios	–	0.38	0.54	0.15	0.25	0.54	0.34
*ε*_IR_	0.52	0.23	0.304 / 0.363	0.3	–	0.26	0.23
Incident angle	30°	40°	50°	45°	–	–	45°
ITO layer	Four	Double	Double	Double	Single	Single	Single
Layout	Identical elements	Identical elements	Identical elements	Identical elements	Deep learning& PSO	Checker-board	Chaotic coding
Method	Absorption	Absorption	Absorption	Absorption	Hybrid	Scattering	Scattering

Given these merits, the proposed chaotic coding metasurface holds promise for practical multispectral stealth applications. For instance, when mounted on the canopy of an unmanned aerial vehicle (UAV) or the window of a military vehicle, it can simultaneously reduce radar detectability and infrared radiation while maintaining optical transparency for vision or sensor systems. Additionally, integration into smart building windows could provide both thermal camouflage and radar invisibility without sacrificing natural lighting [53], which endows the material with great application value in transportation, military, and civilian field.

## Conclusions

In summary, a chaotic paradigm and multi-scale strategy were proposed and experimentally demonstrated to address radar-IR-optical multispectral stealth by using a single-layer coding ITO platform. The multi-scale design concept is realized by integrating millimeter-scale representative elements, centimeter-scale phase-coding subarrays, and decimeter-scale meta-arrays. A quantitative mapping between chaotic initial parameters, and corresponding microwave scattering and IR emissivity characteristics is established, enabling deterministic control over multispectral stealth performance. Theoretically, two-dimensional autocorrelation analysis establishes broadband wavevector diffusion as the physical origin of wide-angle RCS reduction, validating the underlying design paradigm. Numerical and experimental results confirm that our proposed metadevice achieves broadband RCS reduction (> 10 dB) within 8 ~ 18 GHz even at oblique incidence of 45°. Moreover, it maintains low IR emissivity (~ 0.23) and high optical transparency (~ 71.2%). Unlike conventional phase-coding based purely random distributions, the chaotic system introduces tunability of scaling factor α, allowing a flexible balance between IR suppression and microwave scattering diffusion. Overall, our deterministic chaotic coding paradigm establishes a versatile and scalable route for multispectral stealth integration, offering a new platform for lightweight, broadband, and optically transparent stealth metasurfaces and opening new opportunities for applications in integrated photonic systems and thermal management.

## Supplementary Information

Below is the link to the electronic supplementary material.

Supplementary file1 (DOCX 19256 kb)

## References

[1] X. Feng, M. Pu, F. Zhang, R. Pan, S. Wang et al., Large-area low-cost multiscale-hierarchical metasurfaces for multispectral compatible camouflage of dual-band lasers, infrared and microwave. Adv. Funct. Mater. **32**(36), 2205547 (2022). 10.1002/adfm.202205547

[2] R. Zhu, H. Zhu, B. Qin, W. Yao, M. Zhao et al., Digital camouflage encompassing optical hyperspectra and thermal infrared-terahertz-microwave tri-bands. Nat. Commun. **16**, 8112 (2025). 10.1038/s41467-025-63563-3 40885719 10.1038/s41467-025-63563-3 PMC12398538

[3] H. Lin, F. Shen, Z. Zhang, J. Luo, C. Huang et al., Trans-scale hierarchical metasurfaces for multispectral compatible regulation of lasers, infrared light, and microwaves. Nanophotonics **14**(17), 2939–2952 (2025). 10.1515/nanoph-2025-0224 40896154 10.1515/nanoph-2025-0224 PMC12397730

[4] Y. Huang, Y. Zhu, B. Qin, Y. Zhou, R. Qin et al., Hierarchical visible-infrared-microwave scattering surfaces for multispectral camouflage. Nanophotonics **11**(16), 3613–3622 (2022). 10.1515/nanoph-2022-0254 39634446 10.1515/nanoph-2022-0254 PMC11501711

[5] L. Zhao, H. Zhai, Y. Qian, X. Xu, W. Xing et al., Ultralight core–shell polypyrrole@bacterial cellulose aerogel for broadband electromagnetic wave absorption and efficient heat insulation. Carbohydr. Polym. **381**, 125200 (2026). 10.1016/j.carbpol.2026.125200 41943307 10.1016/j.carbpol.2026.125200

[6] M. Dong, L. Zhou, J. Wang, G. Wang, X. Zhang et al., Versatile cellulose nanofiber assisted preparation of magnetic Carbon/MXene aerogel for broadband microwave absorption and infrared stealth. Carbon **252**, 121414 (2026). 10.1016/j.carbon.2026.121414

[7] Y. Zhang, Y. Zhang, Y. Bai, L. Yan, G. Xu et al., Synergistic hollow structure design and defect engineering in dandelion-like α-MnO_2_ for superior radar-infrared compatible camouflage. Adv. Mater. **38**(5), e12477 (2026). 10.1002/adma.202512477 41099127 10.1002/adma.202512477

[8] Z. Xie, Z. Gao, T. Yu, J. Du, J. Qiu, Multifunctional MXene/MOF-derived foam for adaptive radar-infrared stealth and structural sensing. Adv. Funct. Mater. **36**(31), e30008 (2026). 10.1002/adfm.202530008

[9] R. Ji, L. Pan, Z. Xi, J. Yu, K. Liang et al., Combining microwave absorption, thermal insulation, and sensing properties of N-doped C foams@Ni@C@AB silicone elastomers for applications in Radar-infrared compatible stealth and motion monitoring. J. Mater. Sci. Technol. **262**, 285–298 (2026). 10.1016/j.jmst.2025.10.060

[10] J. Kang, X. Kang, S. Liu, H. Jing, J. Wang et al., Multifunctional-hierarchical flexibility metasurfaces for multispectral compatible camouflage of microwave, infrared and visible. Opt. Express **31**(18), 29280 (2023). 10.1364/oe.494367 37710732 10.1364/OE.494367

[11] Y. Cui, J. Wang, H. Sun, Y. Zhu, R. Zhu et al., Visible transparent wideband microwave meta-absorber with designable digital infrared camouflage. Adv. Opt. Mater. **12**(4), 2301712 (2024). 10.1002/adom.202301712

[12] Y. Wang, H. Luo, Y. Shao, H. Wang, T. Liu et al., Detection and anti-detection with microwave-infrared compatible camouflage using asymmetric composite metasurface. Adv. Sci. **11**(43), 2410364 (2024). 10.1002/advs.202410364 10.1002/advs.202410364 PMC11578305 39316364

[13] H.-T. Sun, J. Wang, J. Yang, R.-C. Zhu, J. Zhang et al., Bayesian-neural-network accelerated design of multispectral-compatible camouflage layer with wide-band microwave absorption, customized infrared emission and visible transparency. Mater. Des. **247**, 113400 (2024). 10.1016/j.matdes.2024.113400

[14] J. Ge, X. Wang, B. Li, H. Xue, C. Zhang et al., Optically transparent metasurface with multispectral-compatible camouflage and millimeter-wave transmission window. IEEE Trans. Microw. Theory Tech. **73**(9), 5686–5695 (2025). 10.1109/TMTT.2025.3526833

[15] X. Wang, J. Li, X. Li, Z. Chen, Z. Zhang et al., A wideband microwave absorber compatible with infrared stealth based on the design of magnetic loss layer. IEEE Trans. Microw. Theory Tech. **73**(9), 6109–6121 (2025). 10.1109/TMTT.2025.3549420

[16] H.-R. Zu, B. Wu, B. Chen, W.-H. Li, T. Su et al., Optically and radiofrequency-transparent metadevices based on quasi-one-dimensional surface plasmon polariton structures. Nat. Electron. **6**(7), 525–533 (2023). 10.1038/s41928-023-00995-z

[17] H.-X. Xu, Z. Wang, Y. Wang, Y. Shao, W. Zhang et al., Intelligent metasurface cloak reaches a new plateau: AI-assisted surface engineering for self-adaptive supportive invisibility. Research **9**, 1130 (2026). 10.34133/research.1130 42495010 10.34133/research.1130 PMC13395154

[18] Z.-H. Xu, S. Xu, C. Qian, W. Xu, H. Ren et al., *Chimera* metasurface for multiterrain invisibility. Proc. Natl. Acad. Sci. U. S. A. **121**(6), e2309096120 (2024). 10.1073/pnas.2309096120 38285934 10.1073/pnas.2309096120 PMC10861904

[19] C. Zhang, J. Lou, J. Zhang, Z. Wang, C.-Y. Ji et al., Space-time wavefront synchronized terahertz metasurface. Adv. Mater. **38**(16), e20890 (2026). 10.1002/adma.202520890 41706915 10.1002/adma.202520890

[20] C. Wang, H.-X. Xu, R. Zhu, H. Ding, B. Li et al., 3-D-printed pentahedral polarization-division transmissive metadevice with versatile wavefronts. IEEE Trans. Antennas Propag. **74**(5), 4915–4920 (2026). 10.1109/TAP.2026.3661559

[21] Y. Guo, X. Ma, M. Pu, X. Li, Z. Zhao et al., High-efficiency and wide-angle beam steering based on catenary optical fields in ultrathin metalens. Adv. Opt. Mater. **6**(19), 1800592 (2018). 10.1002/adom.201800592

[22] J. Peng, Y. Zhang, Z. Chen, Q. Wen, S. Wang et al., Annular microfluidic meta-atom fusion-enabled broadband metamaterial absorber. Nano-Micro Lett. **18**(1), 169 (2026). 10.1007/s40820-025-02018-2 10.1007/s40820-025-02018-2 PMC12765792 41486347

[23] H. Wang, B. Zhan, Y. Zhang, Z. Tan, J. Ding et al., Multi-scale synergistic regulation strategy to develop mesoporous carbon hollow nanospheres/bean-shaped nanofibers for corrosion-resistant, flexible, and lightweight microwave absorbers. Research **9**, 1051 (2026). 10.34133/research.1051 41551918 10.34133/research.1051 PMC12805010

[24] Z. Song, J.-F. Zhu, X. Wang, R. Zhang, P. Min et al., Origami metamaterials for ultra-wideband and large-depth reflection modulation. Nat. Commun. **15**, 3181 (2024). 10.1038/s41467-024-46907-3 38609351 10.1038/s41467-024-46907-3 PMC11015009

[25] X. Su, X. Gao, J. Wang, Y. Zhang, Y. Liu et al., Multifunctional broadband electromagnetic wave absorption through structural engineering of hierarchical aerogel-honeycomb metacomposites. Carbon **243**, 120517 (2025). 10.1016/j.carbon.2025.120517

[26] F. Yuan, H.-X. Xu, X.-Q. Jia, G.-M. Wang, Y.-Q. Fu, RCS reduction based on concave/convex-chessboard random parabolic-phased metasurface. IEEE Trans. Anntenas. Propag. **68**(3), 2463–2468 (2020). 10.1109/TAP.2019.2940503

[27] M.K.T. Al-Nuaimi, W.G. Whittow, G.-L. Huang, R.-S. Chen, S.-W. Wong, Wideband radar-cross-section reduction using parabolic phased metasurfaces. IEEE Anntenas. Wirel. Propag. Lett. **22**(7), 1547–1551 (2023). 10.1109/LAWP.2023.3250453

[28] M. Qu, C. Zhang, J. Su, J. Liu, Z. Li, Extremely wideband and omnidirectional RCS reduction for wide-angle oblique incidence. IEEE Trans. Anntenas. Propag. **70**(8), 7288–7293 (2022). 10.1109/TAP.2022.3161309

[29] Z. Gao, C. Xu, X. Tian, J. Wang, C. Tian et al., Ultra-wideband flexible transparent metamaterial with wide-angle microwave absorption and low infrared emissivity. Opt. Express **29**(14), 22108 (2021). 10.1364/oe.428184 34265982 10.1364/OE.428184

[30] Z. Zhang, L. Zhang, Z. Ren, Y. Zhang, T. Hao et al., Multifunctional ultrathin metasurface with a low radar cross section and variable infrared emissivity. ACS Appl. Mater. Interfaces **16**(16), 21109–21117 (2024). 10.1021/acsami.4c01798 38602127 10.1021/acsami.4c01798

[31] H.-X. Xu, S. Ma, X. Ling, X.-K. Zhang, S. Tang et al., Deterministic approach to achieve broadband polarization-independent diffusive scatterings based on metasurfaces. ACS Photonics **5**(5), 1691–1702 (2018). 10.1021/acsphotonics.7b01036

[32] C. Xia, Z. Lu, Y. Zhang, J. Tan, Broadband high optical transparent intelligent metasurface for adaptive electromagnetic wave manipulation. Research **7**, 334 (2024). 10.34133/research.0334 10.34133/research.0334 PMC10927547 38476476

[33] X. Liu, W.J. Padilla, Thermochromic infrared metamaterials. Adv. Mater. **28**(5), 871–875 (2016). 10.1002/adma.201504525 26619382 10.1002/adma.201504525

[34] X. Jiang, H. Yuan, X. He, T. Du, H. Ma et al., Implementing of infrared camouflage with thermal management based on inverse design and hierarchical metamaterial. Nanophotonics **12**(10), 1891–1902 (2023). 10.1515/nanoph-2023-0067 39635145 10.1515/nanoph-2023-0067 PMC11501626

[35] R. Hu, W. Xi, Y. Liu, K. Tang, J. Song et al., Thermal camouflaging metamaterials. Mater. Today **45**, 120–141 (2021). 10.1016/j.mattod.2020.11.013

[36] X. Li, X. Liao, J. Zeng, Z. Yi, X. He et al., Non-volatile tunable multispectral compatible infrared camouflage based on the infrared radiation characteristics of Rosaceae plants. Opto-Electron. Adv. **8**(8), 250031 (2025). 10.29026/oea.2025.250031

[37] H. Zhu, Q. Li, C. Tao, Y. Hong, Z. Xu et al., Multispectral camouflage for infrared, visible, lasers and microwave with radiative cooling. Nat. Commun. **12**, 1805 (2021). 10.1038/s41467-021-22051-0 33753740 10.1038/s41467-021-22051-0 PMC7985314

[38] W. Xi, Y.-J. Lee, S. Yu, Z. Chen, J. Shiomi et al., Ultrahigh-efficient material informatics inverse design of thermal metamaterials for visible-infrared-compatible camouflage. Nat. Commun. **14**, 4694 (2023). 10.1038/s41467-023-40350-6 37542047 10.1038/s41467-023-40350-6 PMC10403604

[39] H. Chu, H. Zhang, Y. Zhang, R. Peng, M. Wang et al., Invisible surfaces enabled by the coalescence of anti-reflection and wavefront controllability in ultrathin metasurfaces. Nat. Commun. **12**, 4523 (2021). 10.1038/s41467-021-24763-9 34312380 10.1038/s41467-021-24763-9 PMC8313714

[40] J. Luo, X. Fang, X. Liu, Z. Wu, Y. Zeng et al., Functional multispectral camouflage strategy based on flexible transparent metamaterial compatible with radiative cooling. Laser Photonics Rev. **19**(12), 2401905 (2025). 10.1002/lpor.202401905

[41] T. Kim, J.-Y. Bae, N. Lee, H.H. Cho, Hierarchical metamaterials for multispectral camouflage of infrared and microwaves. Adv. Funct. Mater. **29**(10), 1807319 (2019). 10.1002/adfm.201807319

[42] Y. Wu, S. Tan, Y. Zhao, L. Liang, M. Zhou et al., Broadband multispectral compatible absorbers for radar, infrared and visible stealth application. Prog. Mater. Sci. **135**, 101088 (2023). 10.1016/j.pmatsci.2023.101088

[43] S. Fang, N. Xu, L. Zhou, T. Wei, Y. Yang et al., Self-assembled skin-like metamaterials for dual-band camouflage. Sci. Adv. **10**(25), eadl1896 (2024). 10.1126/sciadv.adl1896 38896621 10.1126/sciadv.adl1896 PMC11186495

[44] S. Zhong, L. Wu, T. Liu, J. Huang, W. Jiang et al., Transparent transmission-selective radar-infrared bi-stealth structure. Opt. Express **26**(13), 16466 (2018). 10.1364/oe.26.016466 30119477 10.1364/OE.26.016466

[45] C. Zhang, X. Wu, C. Huang, J. Peng, C. Ji et al., Flexible and transparent microwave–infrared bistealth structure. Adv. Mater. Technol. **4**(8), 1900063 (2019). 10.1002/admt.201900063

[46] X. Liu, P. Wang, C. Xiao, L. Fu, H. Zhou et al., A bioinspired bilevel metamaterial for multispectral manipulation toward visible, multi-wavelength detection lasers and mid-infrared selective radiation. Adv. Mater. **35**(41), 2302844 (2023). 10.1002/adma.202302844 10.1002/adma.202302844 37402134

[47] H.-T. Sun, J. Wang, R.-C. Zhu, Z.-T. Chu, X.-M. Fu et al., Noninvasive inset-integrated meta-atom for achieving single-layer metasurface simultaneously with coded microwave reflectivity and digitalized infrared emissivity. Nanophotonics **13**(17), 3113–3122 (2024). 10.1515/nanoph-2024-0098 39634942 10.1515/nanoph-2024-0098 PMC11501719

[48] J. Ge, Y. Wang, Y. Zhang, C. Long, X. Wang et al., Multispectral metasurface for visible transparency, infrared stealth, and mm-wave frequency-multiplexing. Mater. Des. **253**, 113903 (2025). 10.1016/j.matdes.2025.113903

[49] B.-X. Wang, C. Xu, G. Duan, W. Xu, F. Pi, Review of broadband metamaterial absorbers: from principles, design strategies, and tunable properties to functional applications. Adv. Funct. Mater. **33**(14), 2213818 (2023). 10.1002/adfm.202213818

[50] Z. Wang, H. Luo, Y. Cheng, F. Chen, X. Li, Design of optically transparent coded metamaterial based on an indium tin oxide film using deep learning for radar cross-section reduction. ACS Appl. Nano Mater. **7**(20), 23558–23567 (2024). 10.1021/acsanm.4c03822

[51] Y. Wang, G. Wu, Y. Wang, Q. Jia, J. Liu, Single-layer metasurface: Optical transparency, microwave scattering reduction and infrared emissivity decrease. Opt. Mater. **135**, 113380 (2023). 10.1016/j.optmat.2022.113380

[52] B.A. Munk, Frequency Selective Surfaces, Theory and Design (John Wiley & Sons, New York, USA, 2005)

[53] Y. Liang, L. Deng, B. Luo, L. Zhang, K. Tao et al., Flexible, large area preparable phase change PVA/P(ILs-AM)/SSD films for electromagnetic wave absorption and infrared stealth. iScience **28**(5), 112366 (2025). 10.1016/j.isci.2025.112366 40292308 10.1016/j.isci.2025.112366 PMC12032923

