# Supplementary material for: Radar-Infrared Multi-Scale Bi-Stealth via Optically Transparent Chaotic Coding Metasurface
Source: Nanomicro Lett. 2026 Jul 20;18:445. doi: 10.1007/s40820-026-02294-6 (PMC13385293; doi:10.1007/s40820-026-02294-6)
Supplement: Supplementary file 1 — Supplementary file1 (DOCX 19256 kb) [file 40820_2026_2294_MOESM1_ESM.docx]

Supporting Information for

**Radar-Infrared Multi-Scale Bi-Stealth via Optically Transparent Chaotic Coding Metasurface**

Yanzhao Wang,^1^ Yanzhang Shao,^1^ Dan Liu,^1^ Zhixuan Hu, ^1^ Yifei Xu,^1^ Huanhuan Gao,^1^ Xihong Wang^2^, Xiaogang Su^1,3^, Fei Ding^4, *^, and He-Xiu Xu ^1, *^

^1^ Air and Missile Defense College, Air Force Engineering University, Xi’an 710051, P. R. China

^2^ Integrated Circuit Industry College, Wangzheng School of Microelectronics, Changzhou University, Changzhou 213164, P. R. China

^3^ Key Laboratory of Functional Nanocomposites of Shanxi Province, School of Materials Science and Engineering, North University of China, Taiyuan 030051, P. R. China

^4^ School of Electronic Science and Technology, Eastern Institute of Technology, Ningbo, P. R. China

*Corresponding authors. E-mail: [hxxuellen@gmail.com](mailto:hxxuellen@gmail.com) (He-Xiu Xu); <mailto:fding@eitech.edu.cn> (Fei Ding)

**S1 Supplementary Text**

**S1.1 Optical and IR characteristics of representative elements**

Various sheet resistance films are fabricated through an identical process flow, with thickness being the primary variable parameter. The sheet resistance is inversely proportional to the film thickness when the material resistivity remains constant. The specific thickness values corresponding to each target sheet resistance are systematically summarized in the table below, providing a clear reference for process control and performance adjustment.

Table S1 Thickness and Optical Transparency of ITO film for Various Sheet Resistances

| *Rs* (Ω/sq) | Thickness of ITO film (nm) | Optical transparency |
| --- | --- | --- |
| 5 | 260 | 78% |
| 6 | 185 | 87% |
| 15 | 135 | 82% |
| 20 | 65 | 80% |
| 40 | 55 | 82% |
| 60 | 45 | 82% |
| 100 | 23 | 88% |
| 150 | 20 | 88% |
| 200 | 15 | 88% |

To further investigate the IR emissivity of ITO films with different resistance values, IR measurements were conducted using a Nicolet iS50 FT-IR spectrometer and an IR-2 dual-band IR emissivity measurement instrument. Four ITO samples with sheet resistances of 100, 40, 6, and 20 Ω/sq, each measuring 36 ×36 mm^2^, were tested using an IR thermal imager (see Fig. S1). As shown in Fig. S1b, all ITO samples were placed on a heating plate maintained at around 60 °C. As expected, the surface temperature of ITO samples increased with square resistance, indicating that large square resistance results in degraded IR camouﬂage performance (i.e., higher IR emissivity).

To illustrate the relationship between *N* and the unit cell dimension in Fig. 2a more clearly, Table S1 provides a summary of the corresponding side length *w*_n_ and total IR emissivity *ε*_total_ for various *N*.

Table S2 Design parameters *w*_n_ and *ε*_total_ for Different *N* with *p*=12 mm

| *N* | *w*_n_ (mm) | *ε*_total_ | *N* | *w*_n_ (mm) | | *ε*_total_ |
| --- | --- | --- | --- | --- | --- | --- |
| 1 | 11.90 | 0.11 | 16 | | 0.64 | 0.31 |
| 2 | 5.90 | 0.13 | 17 | | 0.60 | 0.32 |
| 3 | 3.90 | 0.14 | 18 | | 0.56 | 0.34 |
| 4 | 2.90 | 0.15 | 19 | | 0.52 | 0.35 |
| 5 | 2.30 | 0.17 | 20 | | 0.49 | 0.36 |
| 6 | 1.90 | 0.18 | 21 | | 0.46 | 0.38 |
| 7 | 1.61 | 0.20 | 22 | | 0.44 | 0.39 |
| 8 | 1.40 | 0.21 | 23 | | 0.41 | 0.40 |
| 9 | 1.23 | 0.22 | 24 | | 0.39 | 0.42 |
| 10 | 1.10 | 0.23 | 25 | | 0.37 | 0.43 |
| 11 | 0.99 | 0.25 | 26 | | 0.35 | 0.44 |
| 12 | 0.90 | 0.26 | 27 | | 0.33 | 0.46 |
| 13 | 0.82 | 0.27 | 28 | | 0.31 | 0.47 |
| 14 | 0.75 | 0.29 | 29 | | 0.30 | 0.48 |
| 15 | 0.70 | 0.30 | 30 | | 0.28 | 0.50 |

To further investigate IR property of ITO, numerical simulations were conducted using CST Microwave Studio, see Fig S2. In the simulation, the dielectric constant *ε*_r_ of ITO was represented by a Drude model with the epsilon infinity *ε*_b_ = 3.95, plasma frequency *ω*_p_ = 3.07×10^15^ s^−1^, and damping constant of *ω*_c_ = 1.82×10^14^ s^−1^, indicating a metal-like behavior in the IR band according to Fig. S2b. According to Kirchhoff’s law of thermal radiation, the emissivity of an object is equal to its absorptivity under thermal equilibrium. Thus, the average emissivity of ITO is calculated to be as low as ⁓0.1 across 3-14 μm, consistent with experimental results. Periodic boundary conditions were applied in both *x* and *y* directions during CST simulations. The simulated IR reflectivity spectra of ITO thin films with various sheet resistances, as well as the corresponding IR emissivity spectra as a function of film thickness, are depicted in Figs. S2c and S2d, respectively. This observation further explains that increased sheet resistance results in higher IR emissivity especially within the critical 8–14 μm IR atmospheric window, thus weakening the IR camouflage performance. The multi-scale arrangement modulates the local electromagnetic response and surface morphology, leading to changes in both the IR absorption and the effective emissivity. Specifically, variations in patch size, spacing, and local density alter the surface plasmon resonance behavior and the coupling to IR radiation, resulting in a non-trivial dependence of emissivity on the multi-scale configuration.

**S1.2 Microwave characteristics of representative elements**

To investigate the influence of thickness on the microwave reflection, Fig. S3 illustrates the variation in reflection amplitudes of the representative element with respect to *N* and frequency under normal incidence of *x*-polarized waves for *h* = 2 mm and *h* = 3 mm. As observed in Fig. S3a and S3b, an increase in thickness leads to a higher overall reflection amplitude and a redshift in the resonant frequency. Furthermore, the frequency bandwidth over which the amplitude exceeds 0.9 becomes progressively wider with the increased thickness. Physically, an increased substrate thickness elevates the effective resonant cavity height and strengthens electromagnetic field confinement within the dielectric substrate, which reduces the resonant wavevector and consequently leads to resonant frequency redshift. In addition, the thickened substrate enhances modal coupling between the patterned ITO structure and substrate eigenmodes, enriches the resonant response modes, and thereby broadens the reflection operating bandwidth.

Due to the symmetric square patch configuration of the unit cells, the design exhibits excellent rotational symmetry and thus demonstrates negligible polarization dependence. Consequently, under normal incidence, the amplitude responses for both *y*- and *x*- polarizations are nearly identical. The resonant unit denotes the fundamental building block of a metasurface. Here, an ITO square patch which supports localized electromagnetic resonances at specific frequencies is regarded as the resonant unit. Its resonant properties are dominated by geometric parameters, particularly the thickness *h*. To characterize the resonance of the representative element composed of ITO patches, the amplitude-frequency characteristic (S_11_) and surface current distribution are displayed in Fig. S3c-d and Fig. S4. It can be observed from Fig. S3c-d that as the oblique incident angle increases, the resonance strength deepens gradually under *y*-polarization, whereas the resonant frequency exhibits an obvious shift under *x*-polarization. The strengthened resonance gives rise to enhanced resonant absorption, which directly reduces the reflected electromagnetic signal, leading to a lower reflection amplitude at larger incident angles. On the whole, a stable reflection coefficient over 8~18 GHz can be ensured within incident angle up to 45°, which meets the targeted design requirements. The surface current distributions illustrated in Fig. S4 reveal the currents in upper and lower layers exhibit opposite directions, thus constructing a closed current loop, which further validates the generation of a circulating magnetic field surrounding ITO patches of representative elements.

**S1.3 Derivation of the ECM**

The values of *C*₁ (capacitance) and *L*₁ (inductance) were extracted using the empirical formulas in [S1]. The methodology is outlined as follows:

$B=\frac{2(\varepsilon_{r}+1)l}{Z_{0}\lambda}\left\{\ln[\csc\frac{\pi(P-l)}{2P}]+\frac{1}{2}(1-\beta^{2})^{2}\frac{2A(1-\frac{\beta}{4}^{2})+4A^{2}\beta^{4}}{(1-\frac{\beta}{4}^{2})+2A\beta^{2}(1+\frac{\beta}{2}^{2}-\frac{\beta}{8}^{4})+2A^{2}\beta^{6}}\right\}$ （S1）

$X=\frac{Z_{0}l}{\lambda}\left\{\ln[\csc\frac{\pi l}{2P}]+\frac{1}{2}(1-\beta^{2})^{2}\frac{2A(1-\frac{\beta}{4}^{2})+4A^{2}\beta^{4}}{(1-\frac{\beta}{4}^{2})+2A\beta^{2}(1+\frac{\beta}{2}^{2}-\frac{\beta}{8}^{4})+2A^{2}\beta^{6}}\right\}$ （S2）

where $A=1/\sqrt{1-(\frac{P}{\lambda})^{2}}-1$, $\beta=\sin\left[\frac{\pi(P-l)}{2P}\right]$ for $C=B/\omega$, and $\beta=\sin\frac{\pi l}{2P}$ for $L=X/\omega$.

To provide more details, we summarized the corresponding ECM parameters, including inductance *L*_1_ and capacitance *C*_1_, for varied *N* at a fixed period of *p*=12 mm in Table S3.

Table S3 ECM parameters *L*_1_ and *C*_1_ for Different *N* with *p*=12 mm

| *N* | *L*_1_ (pH) | *C*_1_ (pF) | *N* | *L*_1_ (pH) | | *C*_1_ (pF) |
| --- | --- | --- | --- | --- | --- | --- |
| 1 | 0.204 | 0.324 | 16 | | 45.968 | 0.091 |
| 2 | 0.808 | 0.246 | 17 | | 51.442 | 0.088 |
| 3 | 1.806 | 0.216 | 18 | | 57.168 | 0.083 |
| 4 | 3.180 | 0.192 | 19 | | 63.137 | 0.080 |
| 5 | 4.930 | 0.175 | 20 | | 69.340 | 0.076 |
| 6 | 7.038 | 0.162 | 21 | | 75.768 | 0.074 |
| 7 | 9.499 | 0.154 | 22 | | 82.434 | 0.070 |
| 8 | 12.304 | 0.144 | 23 | | 89.286 | 0.067 |
| 9 | 15.444 | 0.135 | 24 | | 96.360 | 0.062 |
| 10 | 18.900 | 0.130 | 25 | | 103.625 | 0.060 |
| 11 | 22.682 | 0.121 | 26 | | 111.072 | 0.057 |
| 12 | 26.760 | 0.113 | 27 | | 118.692 | 0.056 |
| 13 | 31.135 | 0.107 | 28 | | 126.476 | 0.053 |
| 14 | 35.798 | 0.102 | 29 | | 134.415 | 0.052 |
| 15 | 40.740 | 0.096 | 30 | | 142.530 | 0.048 |

The reflection coefficient *Γ* can be derived from *Γ* = (*Z*_in_ − Z₀) / (*Z*_in_ + Z₀), where *Z*_in_ = *R*₁ + j(*ωL*₁) − j(*ωC*₁)^-1^ represents the equivalent input impedance of the representative element, and Z₀ = 377 Ω denotes the free-space impedance. The reflection phase *φ* is also related to *Z*_in_ by *φ=*arg(*Γ*). According to Table S3, *L*_1_ rises while *C*_1_ declines with the increase of parameter *N*. The reactive component of input impedance increases accordingly, leading to a larger impedance magnitude and enhanced inductive characteristic of Z_in_.

**S1.4 The** **robustness of chaotic coding metasurfaces**

A comparative analysis of microwave RCS performance and computational efficiency across multiple optimization algorithms is presented. Compared with purely random distributions, chaotic sequences are fully reproducible for given initial conditions, which directly tune the scaling factor *α* and thus both microwave and IR responses. Results indicate that the chaotic algorithm significantly outperforms other algorithms in terms of computational efficiency. This advantage becomes increasingly pronounced as the number of subarray increases—attributable to the algorithm’s deterministic nature, which eliminates stochastic search behavior and enhances convergence speed. Furthermore, the chaotic algorithm exhibits strong robustness with respect to initial conditions, ensuring consistent performance across different starting points.

Herein, we provide a theoretical interpretation of the chaotic coding mechanism. The far-field scattering pattern of a metasurface is closely related to the Fourier transform of its reflection-coefficient distribution ,

（S3）

where *L*^2^ represents the total area of metasurface. To achieve optimal wave diffusion, the phase distribution *φ*(*x*, *y*) should be designed such that the scattered field becomes as uniform as possible across all directions. In general, the more spatially random the metasurface structure, the broader its Fourier transform (i.e., the wavevector spectrum). Therefore, the goal is to generate a specific distribution whose energy is uniformly distributed over the entire wavevector space. Based on the Wiener–Khinchin theorem, the far-field radiation power pattern can be represented as the Fourier transform of the spatial autocorrelation function associated with *R*(*x*, *y*). To realize uniform scattering, the reflection coefficient distribution must possess perfect randomness, with the reflection coefficients at any two distinct positions being mutually uncorrelated. The metasurface generated by chaotic coding method exhibit ideal autocorrelation properties, as illustrated in Fig. S5. The autocorrelation function of the chaotic coding matrix reveals its inherent randomness and aperiodicity. Precisely this characteristic—featuring a central sharp peak accompanied by rapidly decaying random sidelobes (as shown in Fig. S5c)—enables chaotic coding to realize a uniform wavevector spatial distribution, thereby achieving wide-angle RCS reduction. In contrast, the autocorrelation function of checkerboard metasurface exhibits periodic sharp peaks in space, resulting in intense reflection peaks along specific directions and thus failing to achieve uniform scattering. Herein, the two-dimensional autocorrelation function is calculated via fast fourier transform.

Based on above analysis, the scattering uniformity can be further improved by increasing the coding length. Specifically, a larger electrical size of the metasurface (i.e., more coding elements) leads to a higher frequency resolution in the Fourier transform, making the scattering background closer to an ideal continuous uniform distribution. To further validate the scalability of the proposed approach, 2-bit and 3-bit chaotic metasurfaces were designed, and their phase distributions and theoretical scattering patterns are shown in Fig. S6.

It is evident that electromagnetic waves scatter in all directions, which facilitates stealth effects in the microwave band. By identifying appropriate element structures and arranging them according to the phase distribution illustrated in Figs. S6a and S6b, effective microwave stealth performance can be achieved. These results demonstrate the broad applicability of our proposed chaotic coding strategy.

To demonstrate the robustness of our proposed strategy, Fig. S7 illustrates the configuration diagrams and corresponding theoretical simulated scattering patterns for the extended metasurface arrays consisting of 20×20 and 30×30 subarrays, respectively. It is clearly seen that increasing the array area gives rise to more significant energy dispersion, thereby yielding enhanced microwave stealth effectiveness.

**S1.5 The properties of different materials**

Table S4 Thickness and Optical Transparency of PVC film with different thickness

| No. | Thickness of PVC film (mm) | Optical transparency |
| --- | --- | --- |
| 1 | 1 | 90.5% |
| 2 | 2 | 91.2% |
| 3 | 3 | 90.9% |

Fig. S8 displays the transmittance of PVC samples with different thicknesses. The optical transmittance values remain approximately 91% for all cases. The 1 mm-thick sample in Fig. S5a tends to bend easily owing to its small thickness, which introduces minor errors in the transmittance measurement. Nevertheless, the overall transmittance stays above 90%, demonstrating the favorable optical transparency of PVC material.

Table S5 Multispectral stealth performance of different materials

| Materials | Typical Materials | Microwave Modulation Capability | IR Emissivity Modulation Capability | Visible Light Transmittance | Comprehensive Performance |
| --- | --- | --- | --- | --- | --- |
| Transparent Conductive Oxide | FTO/AZO | Good | Medium | Medium | Midum |
| Pure Metals | Au/Ag/Al | Poor | Good | Poor | Poor |
| Traditional Semiconductors | Si/GaAs | Medium | Medium | Poor | Poor |
| This work | ITO | Good | Good | Good | Good |

As shown in the table, pure metals show favorable infrared emissivity modulation but poor performance in microwave modulation and visible light transmittance. Traditional semiconductors (Si, GaAs) deliver moderate modulation capability for microwaves and IR radiation, while their visible light transmittance is unsatisfactory. Conventional transparent conductive oxides (FTO, AZO) only achieve medium performance across all indicators. In comparison, the ITO material adopted in this work possesses excellent microwave modulation, infrared emissivity regulation and high visible light transmittance. It outperforms other materials in overall performance and is highly suitable for microwave-infrared-visible compatible stealth applications.

**S1.6 Wideband Bistatic RCS Reduction Performance**

The experimental setup for bistatic measurement configuration under oblique incidence is shown in Fig. S9. Fig. S10 further compares the measured far-field bistatic RCS of the fabricated metadevice and a metal plate at 8 GHz ~ 18 GHz under *x*-polarized incidence. At all three frequencies, the metadevice exhibits significantly lower RCS than the metal plate across nearly the entire angular range, with the most pronounced reduction observed around the specular direction. These results confirm that the chaotic coding method achieves effective and broadband bistatic RCS reduction by efficiently diffusing the incident energy into diffuse, non-specular directions.

**S1.7 Mechanical reliability**

To investigate the mechanical stability and practical applicability of the proposed metasurface, we conducted studies under both bending and damage scenarios, as illustrated in Figs. S11-S13. First, a conformal cylindrical surface was fabricated as shown in Fig. S11a. A copper sheet was attached to the surface as a reference (Fig. S11b), and the proposed conformal sample was then applied onto the same substrate, as depicted in Fig. S11c. The measured RCS reduction is presented in Fig. S11d, which clearly demonstrates significant radar RCS reduction performance.

Furthermore, considering the application scenario on transparent substrates, a curved glass sample (length: 40 cm, width: 34.3 cm, height: 3.4 cm) was prepared. As shown in Fig. S12, the conformal sample exhibits excellent optical transparency while maintaining effective radar stealth capability, thus proving its promising potential for future practical applications.

For further investigation of radar stealth performance after mechanical damage, three different damage cases were introduced in Fig. S13. After friction, the most significant impact is the removal of the ITO layer. Therefore, three different cases were simulated to verify this effect. Edge damage, central damage and scattered small damages are presented in Fig S13. a–c. The RCS curves in Fig. S13 d indicate that different damage positions and sizes exert distinct effects on the stealth performance. Central damage leads to the most obvious performance deterioration, while edge damage has minimal impact. The structure still retains acceptable stealth performance under partial damage conditions.

**S1.8 Thermal image of outdoor experiment**

Fig. S14 presents the IR thermal images at different times during the outdoor experiment. It is obvious that the surface temperature of the camouflage raincoat drops after being covered by the metasurface. Moreover, the temperature also varies gradually over time, confirming the dynamic thermal response of the system under natural environmental conditions.

**S2 Supporting Figures**


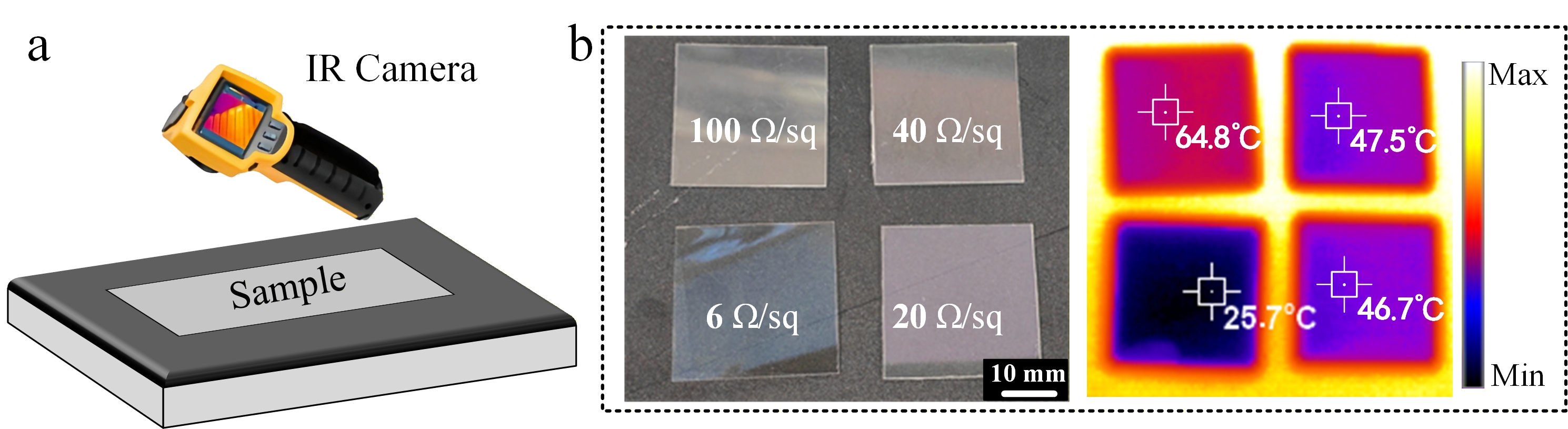


**Fig. S1** (a) Schematic diagram of the IR testing setup. (b) ITO samples with identical dimensions but different square resistance values, along with their corresponding IR thermal images.


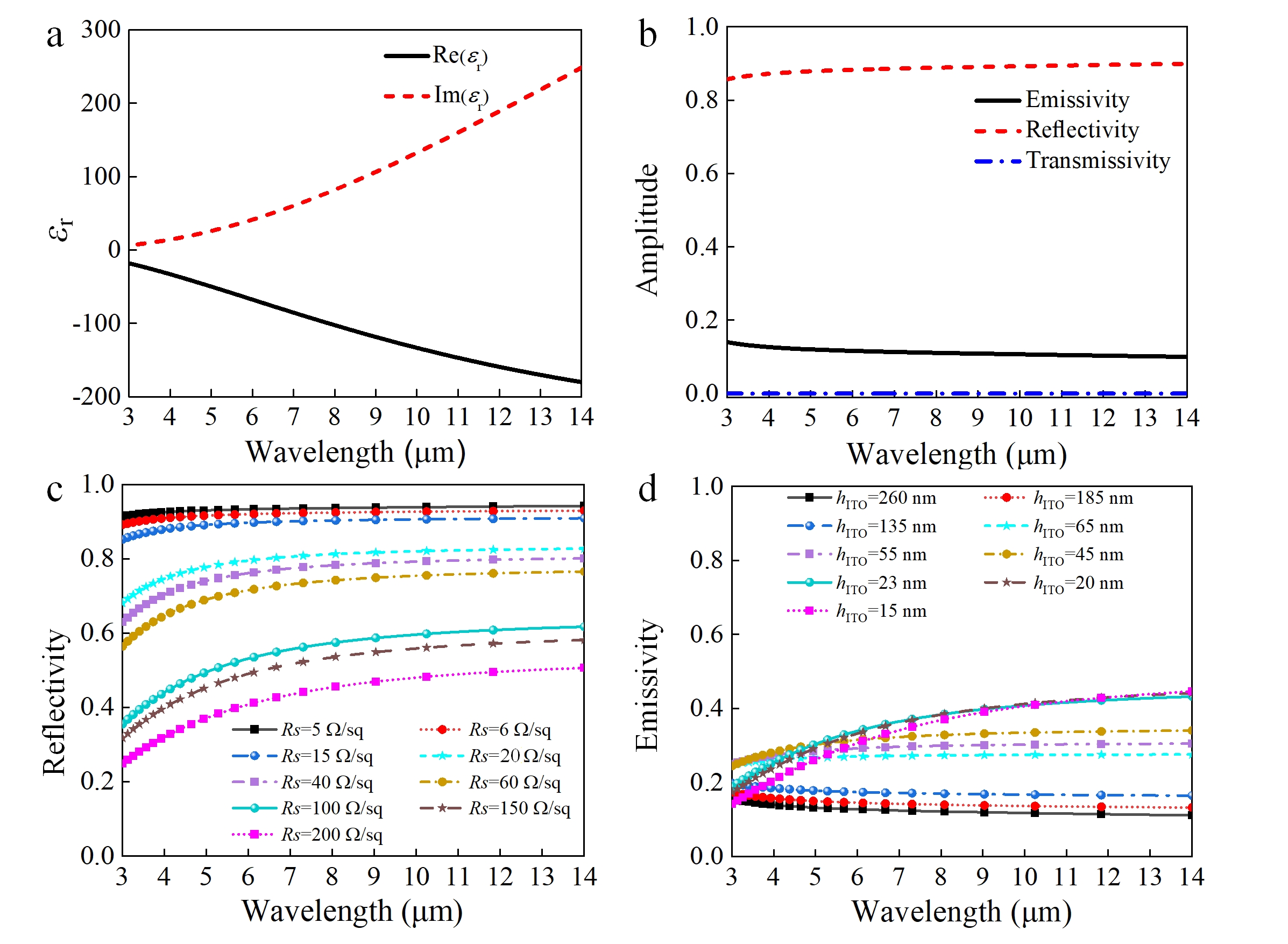


**Fig. S2** (a) Permittivity of ITO across 3–14 μm. (b) Simulated IR emissivity, transmissivity, and reﬂectivity of ITO film with a thickness of 185 nm. (c) Simulated IR reflectivity of ITO film with different sheet resistances and (d) corresponding IR emissivity spectra as a function of film thickness.


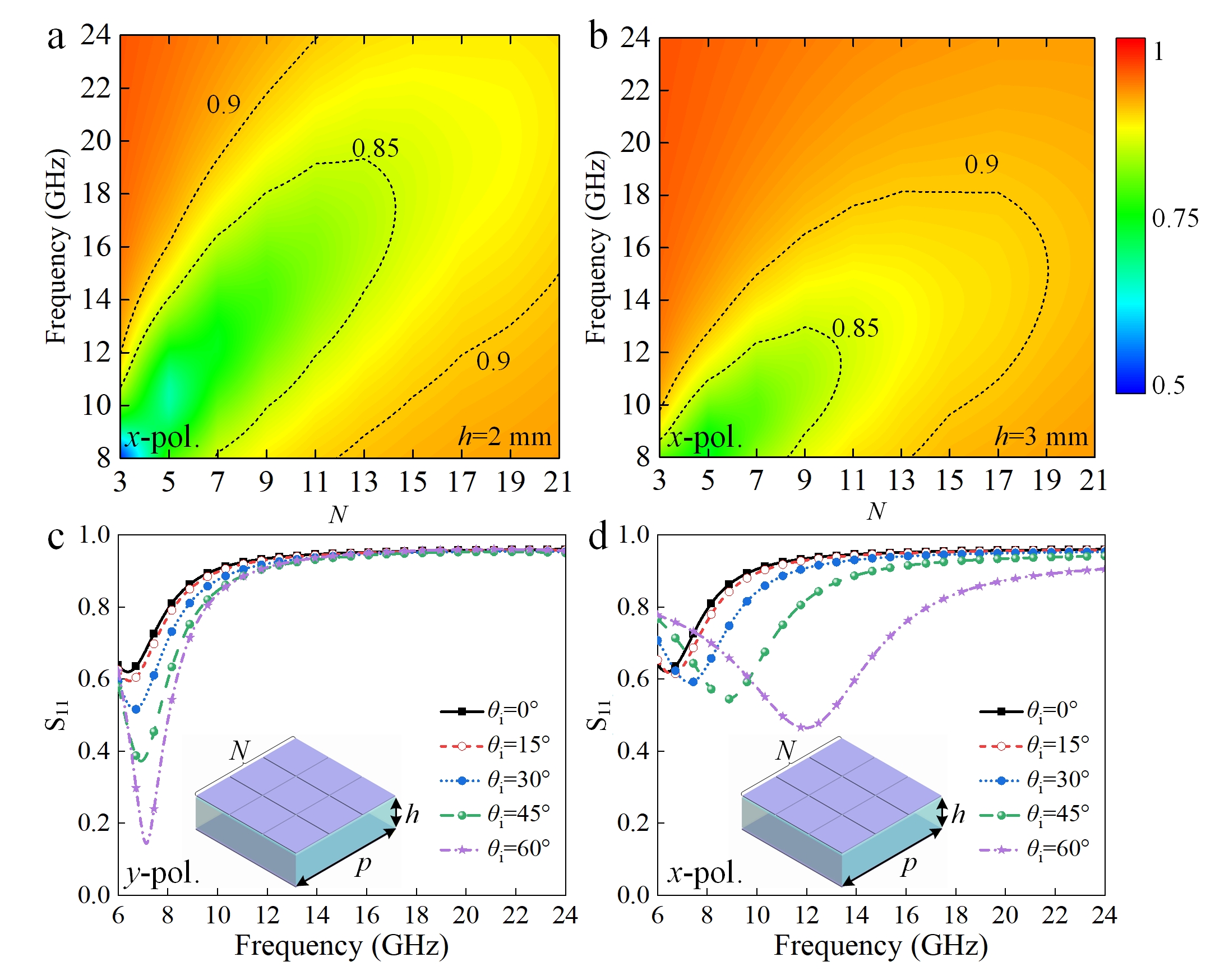


**Fig. S3** Microwave reflection amplitude of representative elements with *N* ranging from 3 to 21 at (a) *h* = 2 mm and (b) *h* = 3 mm under *x*-polarization. Simulated S_11_ of (c) *y*- and (d) *x*-polarizations under different angles within 6~24 GHz.


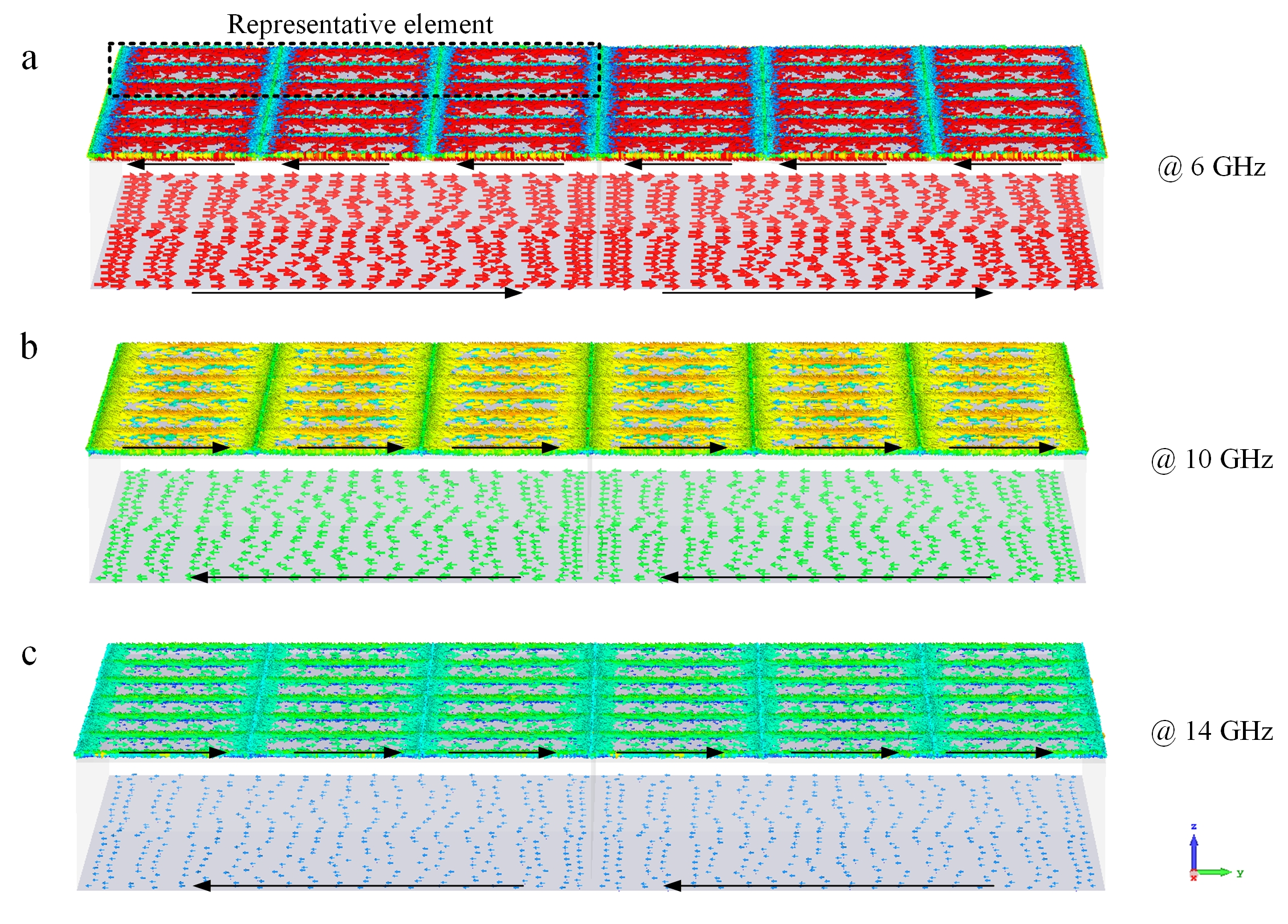


**Fig. S4** Surface current distribution of representative elements at (a) 6, (b) 10 and (c) 14 GHz.


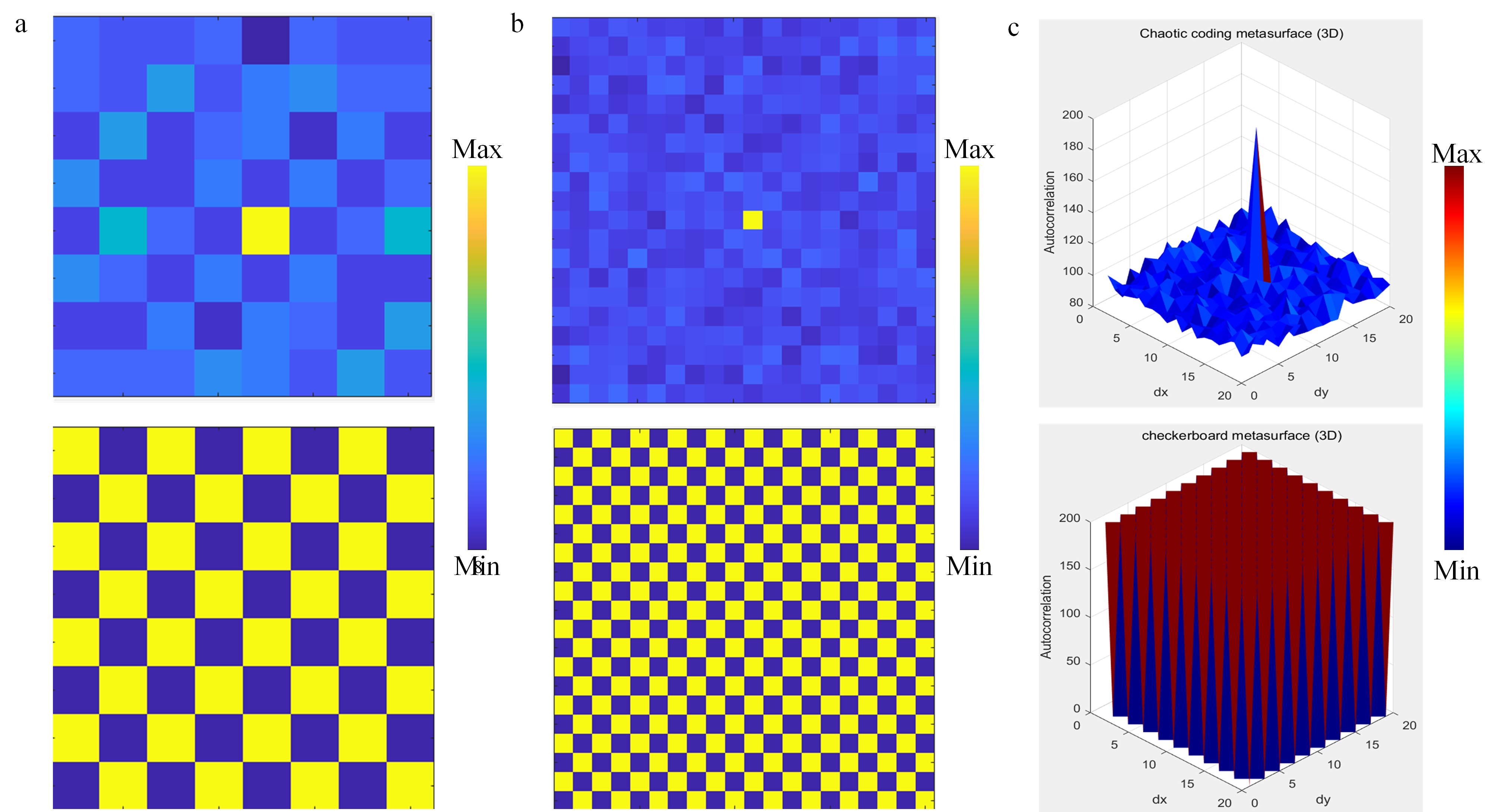


**Fig. S5** Characterization of the two-dimensional autocorrelation of chaotic coding and checkerboard metasurfaces. Autocorrelation for metasurfaces composed of (a) 8×8 and (b, c) 20×20 subarrays based on the chaotic coding method (upper panel) and checkerboard metasurfaces (bottom panel).


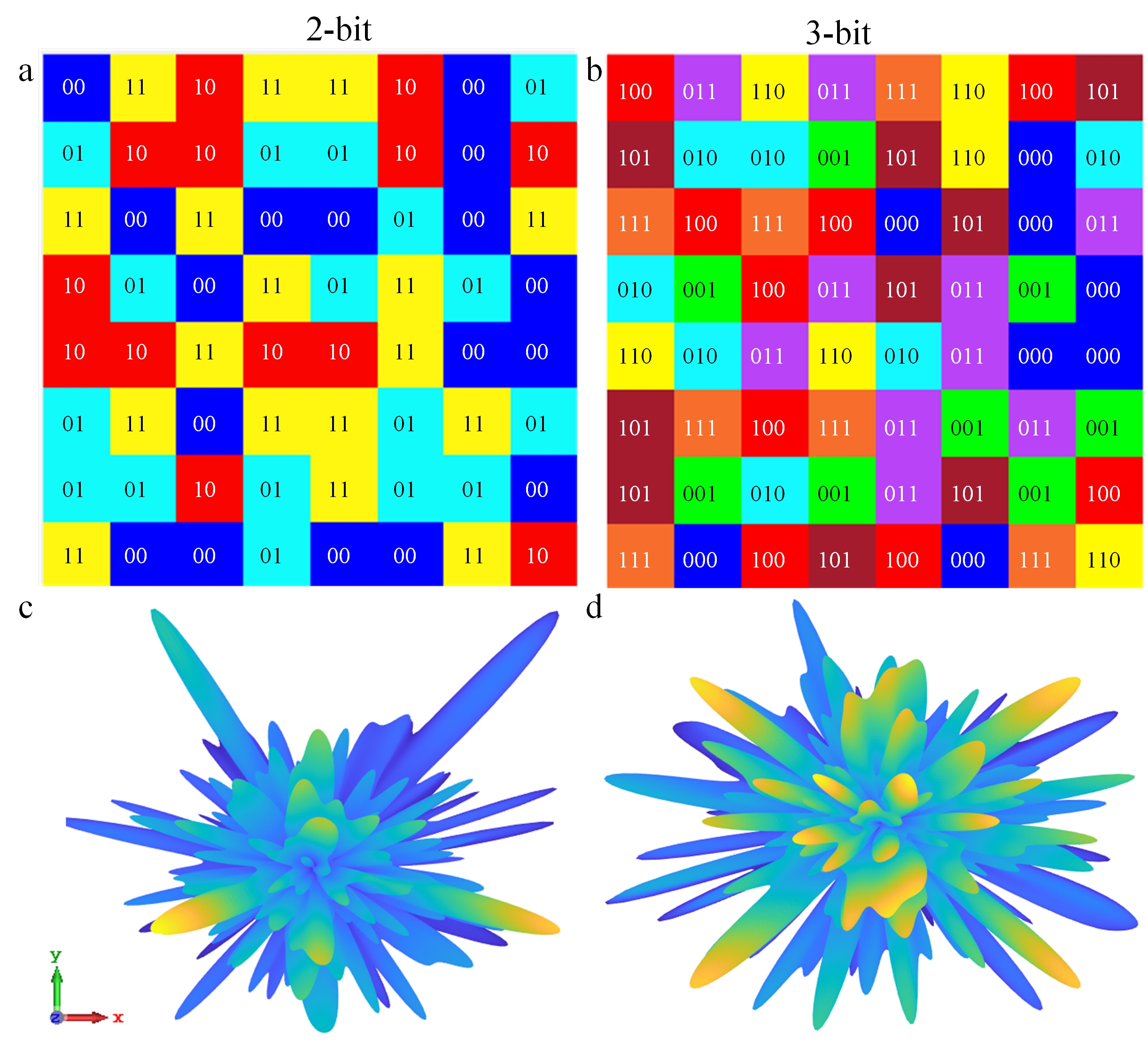


**Fig. S6** (a-b) Phase distributions and corresponding (c-d) scattering patterns of (a, c) 2-bit and (b, d) 3-bit metasurfaces based on the chaotic coding method.


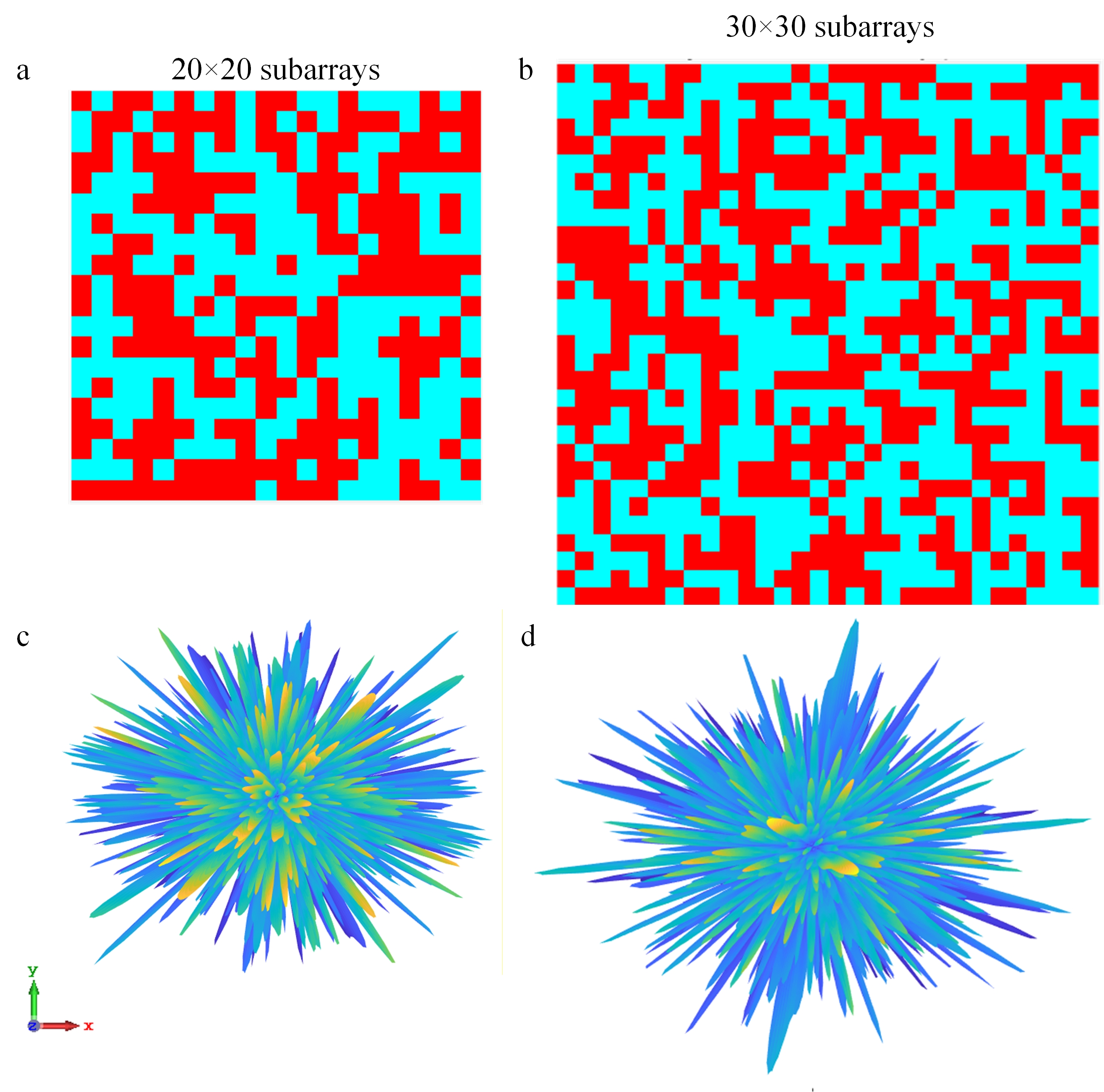


**Fig. S7** (a-b) Phase distributions and corresponding (c-d) scattering patterns of 1-bit coding metasurfaces composed of (a, c) 20×20 and (b, d) 30×30 subarrays based on the chaotic coding method.


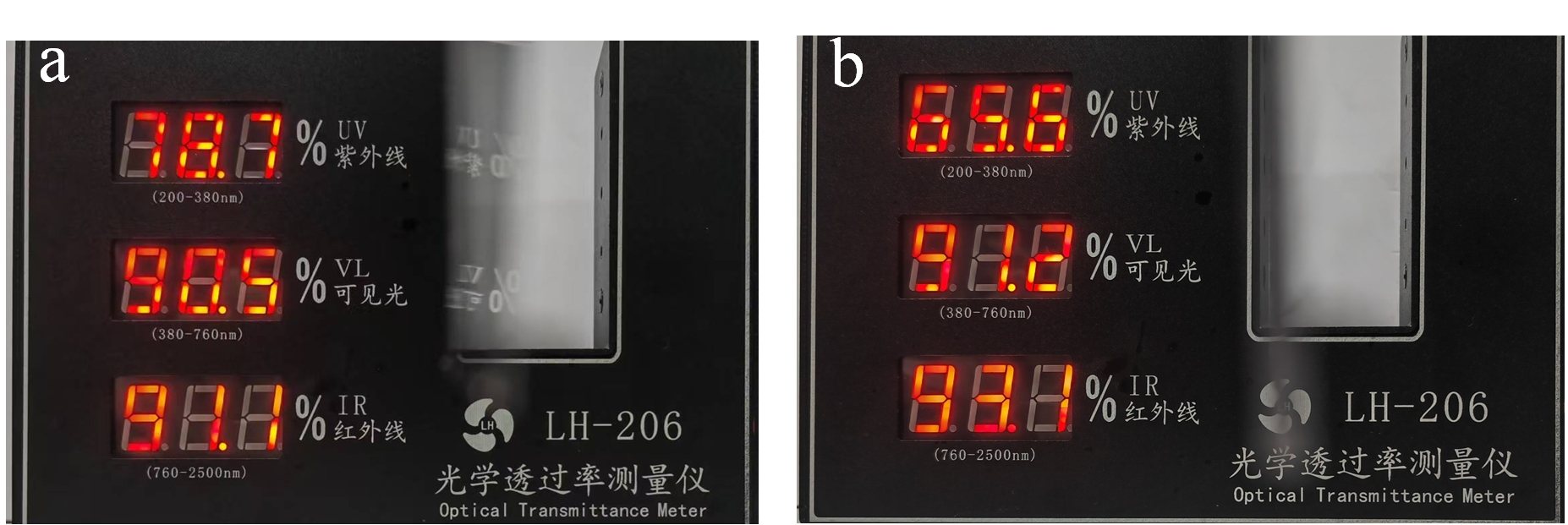


**Fig. S8** Measured optical transmittance of PVC with (a) 1 mm and (b) 2 mm.


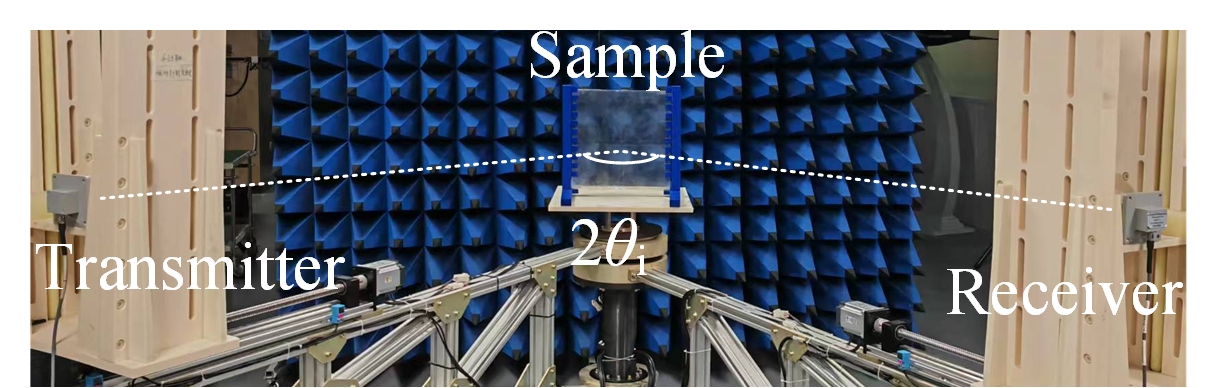


**Fig. S9** Experimental setup for bistatic measurement configuration.


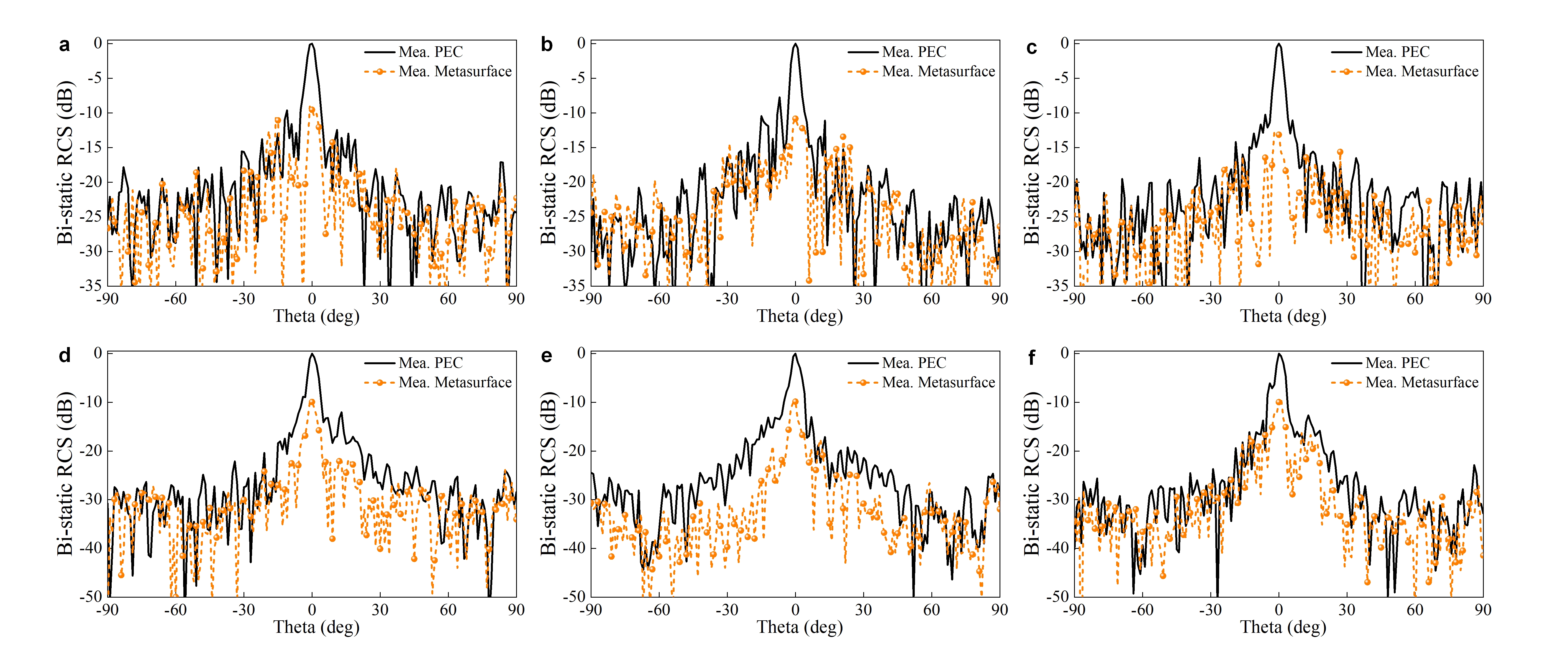


**Fig. S10** Comparisons of the measured far-field bistatic RCS results of the proposed metasurface and a metal plate reference at (a) 8 GHz, (b) 10 GHz, (c) 12 GHz, (d)14 GHz, (e) 16 GHz and (f) 18 GHz under *x*-polarized incidence.


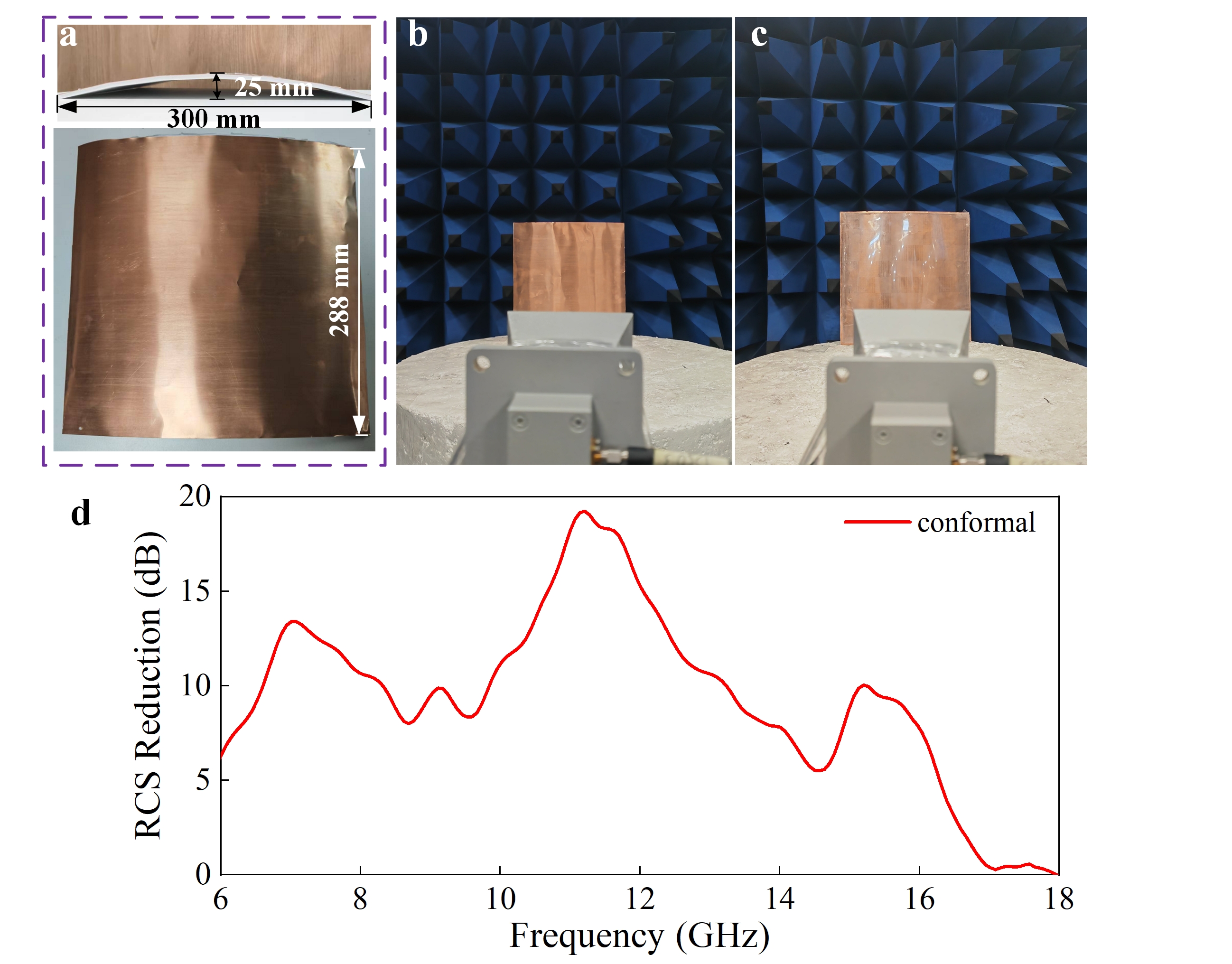


**Fig. S11** Fabricated samples and measured RCS reduction. (a) Curved copper plate and conformal sample. RCS measurement setups for (b) bare and (c) coated plates. (d) RCS reduction of the conformal bending sample.


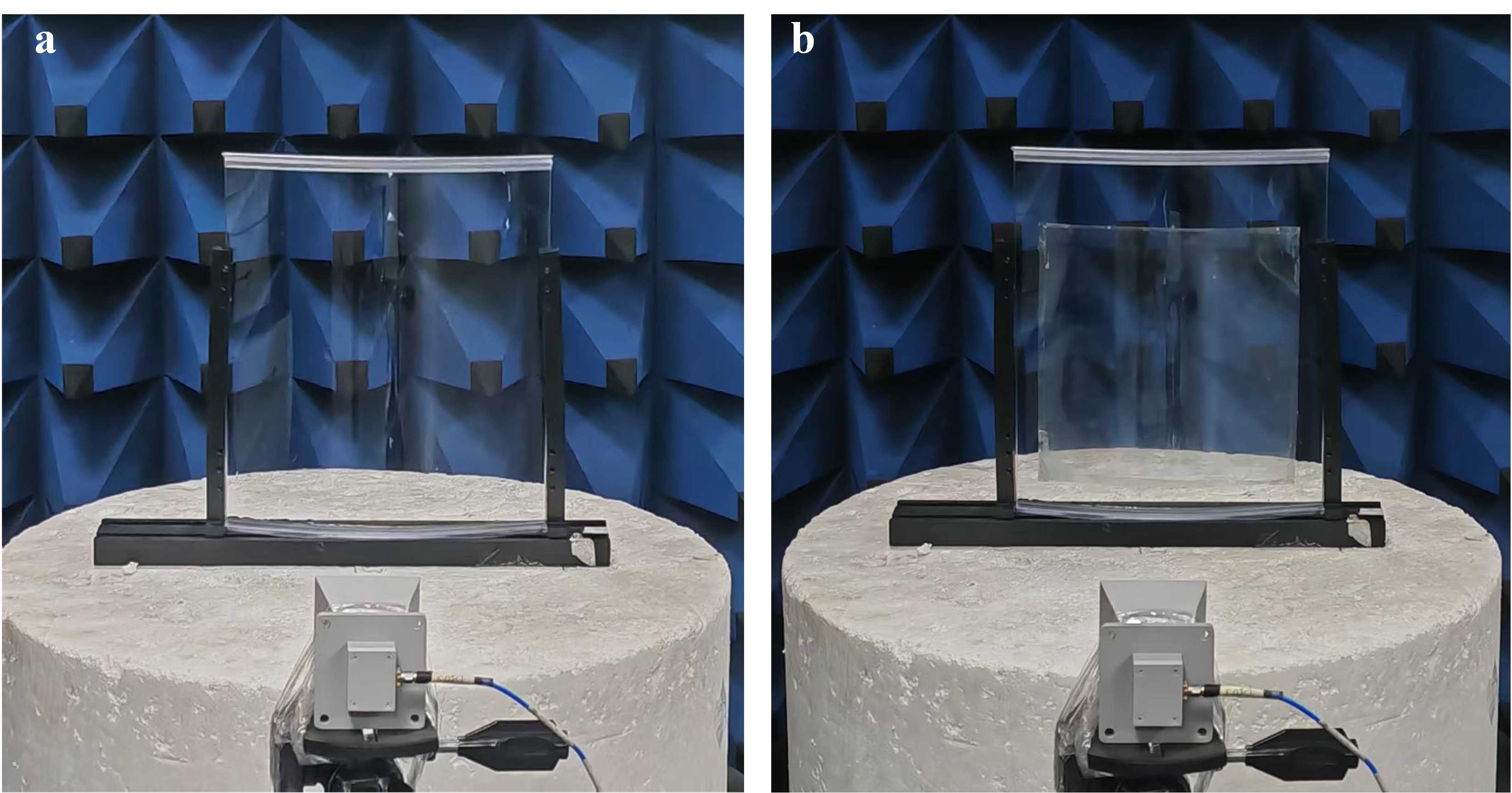


**Fig. S12** Potential application scenarios. (a) Original curved glass sample. (b) Conformal stealth glass integrated with coated metasurface.


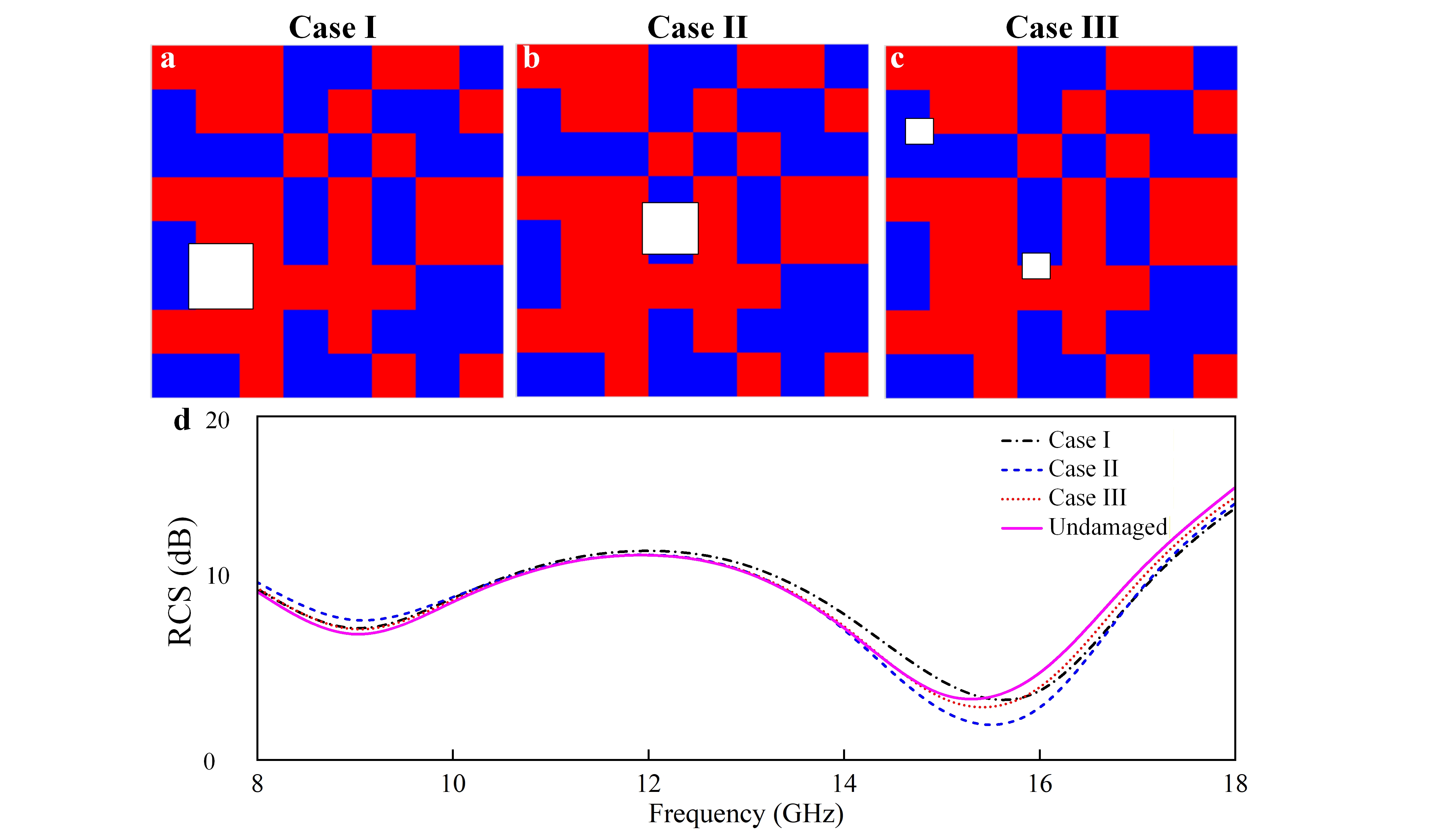


**Fig. S13** Three damage scenarios of the metasurface and corresponding stealth performance. (a) Case I of edge damage. (b) Case II of central damage. (c) Case III of scattered small damages. (d) Comparison of RCS between the undamaged structure and three damaged cases from 8 ~ 18 GHz.


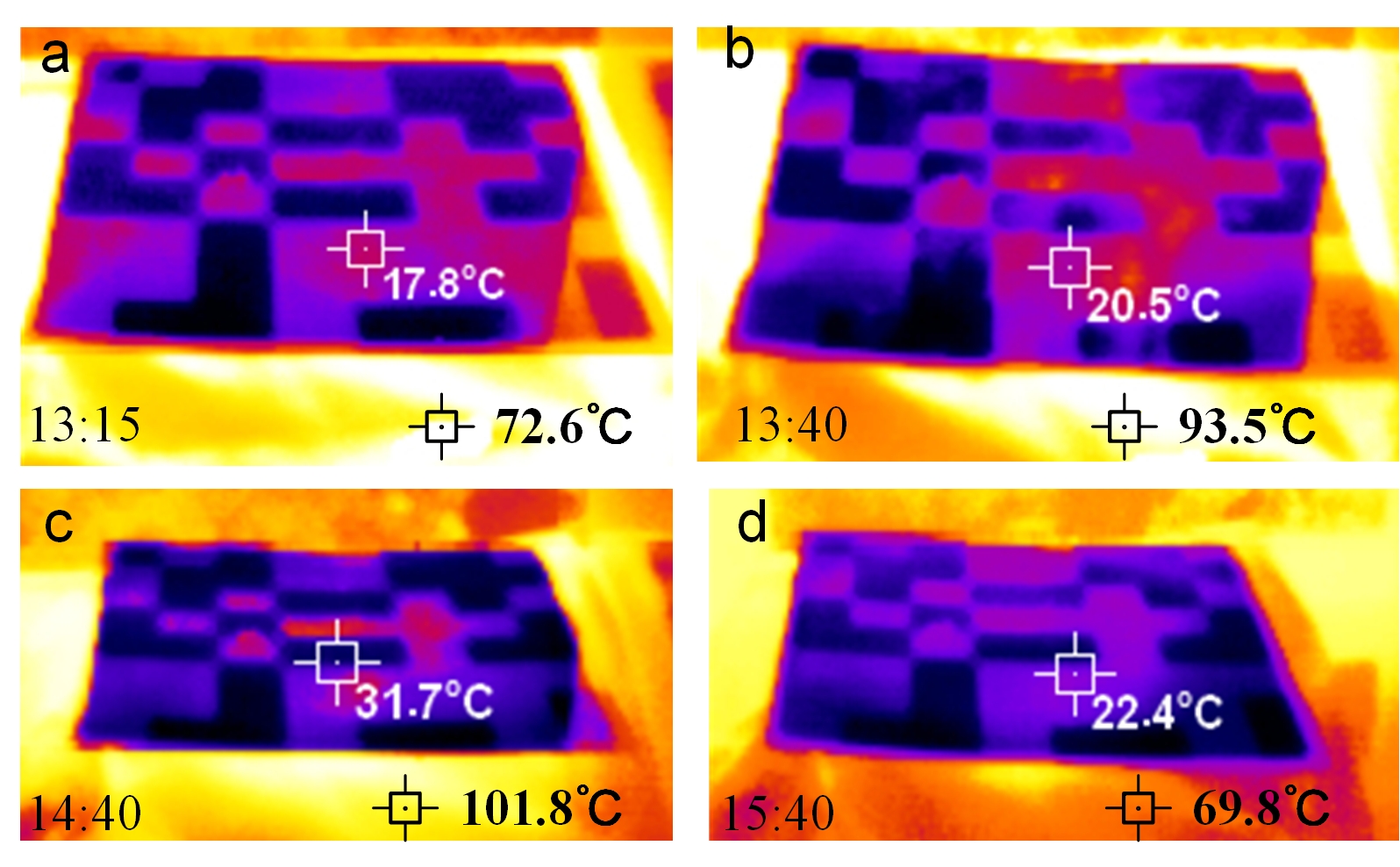


**Fig. S14** IR thermal images of the metasurface and camouflage raincoat captured at (a) 13:15, (b) 13:40, (c) 14:40 and (d) 15:40.

**Supplementary References**

[S1] B.A. Munk, Frequency Selective Surfaces, Theory and Design (John Wiley & Sons, New York, USA, 2005).
